# Rational Design of Benzylidenehydrazinyl-Substituted Thiazole Derivatives as Potent Inhibitors of Human Dihydroorotate Dehydrogenase with *in Vivo* Anti-arthritic Activity

**DOI:** 10.1038/srep14836

**Published:** 2015-10-07

**Authors:** Shiliang Li, Guoqin Luan, Xiaoli Ren, Wenlin Song, Liuxin Xu, Minghao Xu, Junsheng Zhu, Dong Dong, Yanyan Diao, Xiaofeng Liu, Lili Zhu, Rui Wang, Zhenjiang Zhao, Yufang Xu, Honglin Li

**Affiliations:** 1 State Key Laboratory of Bioreactor Engineering, Shanghai Key Laboratory of New Drug Design, School of Pharmacy, East China University of Science and Technology, Shanghai 200237, China

## Abstract

Human dihydroorotate dehydrogenase (*h*DHODH) is an attractive therapeutic target for the treatment of rheumatoid arthritis, transplant rejection and other autoimmune diseases. Based on the X-ray structure of *h*DHODH in complex with lead compound **7**, a series of benzylidenehydrazinyl-substituted thiazole derivatives as potent inhibitors of *h*DHODH were designed and synthesized, of which **19** and **30** were the most potent with IC_50_ values in the double-digit nanomolar range. Moreover, compound **19** displayed significant anti-arthritic effects and favorable pharmacokinetic profiles *in vivo*. Further X-ray structure and SAR analyses revealed that the potencies of the designed inhibitors were partly attributable to additional water-mediated hydrogen bond networks formed by an unexpected buried water between *h*DHODH and the 2-(2-methylenehydrazinyl)thiazole scaffold. This work not only elucidates promising scaffolds targeting *h*DHODH for the treatment of rheumatoid arthritis, but also demonstrates that the water-mediated hydrogen bond interaction is an important factor in molecular design and optimization.

Human dihydroorotate dehydrogenase (*h*DHODH) is a mitochondrial inner membrane anchored enzyme that belongs to the class 2 DHODH family of enzymes^1^. It is the fourth enzyme in the *de novo* pyrimidine nucleotide biosynthesis pathway. In humans, resting or fully differentiated cells fulfil their metabolic requirement for pyrimidines through a salvage pathway, whereas activated lymphocytes and other rapidly proliferating tumor cells primarily depend on *de novo* pyrimidine biosynthesis to support their enhanced growth rate^2^,^3^. Inhibition of *h*DHODH can block this *de novo* pyrimidine nucleotide biosynthesis pathway, and reducing the survival of these proliferating lymphocytes and tumor cells. Consequently, *h*DHODH is an attractive target for the treatment of cancer and autoimmune diseases such as rheumatoid arthritis, psoriasis and multiple sclerosis^4^,^5^,^6^.

Clardy *et al*. first solved the three-dimensional crystal structures of *h*DHODH in complex with A771726 (**1**), the active metabolite of leflunomide (**2**), and a brequinar analog, providing the first insights into the specific structural features of class 2 DHODH enzymes. *h*DHODH is composed of two domains connected by an extended loop: a large α/β barrel C-terminal domain and a small N-terminal domain containing two α-helices^7^. The C-terminal domain offers binding sites for FMN and DHO, while the N-terminal domain houses the ubiquinone-binding site^8^. Several anti-tumor and immunosuppressive molecules have been reported to target *h*DHODH at the ubiquinone-binding site^1^,^9^,^10^,^11^. The two best-studied examples (Fig. 1) are brequinar (**3**)^12^ and leflunomide (**2**)^13^,^14^,^15^. The latter, which is the prodrug of the active metabolite A771726 (**1**), has been approved by the FDA for the treatment of rheumatoid arthritis. Unfortunately, both compounds are associated with various side effects. Brequinar was used for the treatment of cancer and graft-versus-host disease on the basis of satisfactory efficacy in animal models but failed in clinical trials due to its narrow therapeutic window^16^. Leflunomide has a disadvantageously long half-life of approximately two weeks in patients and has many side effects, such as symptomatic liver damage and interstitial lung disease^17^. Consequently, the identification of novel potent inhibitors of *h*DHODH as therapeutic agents that inhibit pyrimidine biosynthesis for the treatment of cancer and autoimmune diseases remains of considerable interest^1^,^9^.

Water plays a crucial role in molecular recognition and molecular association^18^. A comprehensive study of water molecules at protein-ligand interfaces was performed by Wang *et al*. in 2007^19^. A total of 1829 ligand-bound water molecules, of which more than half bridged ligand and protein atoms by polar interactions, were identified in the crystal structures of 392 high-resolution protein-ligand complexes. The formation of hydrogen bond networks is specific features of water molecules in active sites^20^. According to Essex *et al*., a tightly bound water molecule can form three or four hydrogen bonds with the protein and the ligand, contributing to a stronger negative binding free energy compared to a loosely bound water molecule, which makes less than three hydrogen bonds^21^. Water molecules have been confirmed to be essential mediators of binding affinity or binding specificity in several ligand-protein systems. Recently, Boxer and Levinson shed light on the significance of water molecules at the structural interface in inhibitor recognition by demonstrating that two structural water molecules in the active site assisted the clinical kinase inhibitor bosutinib in recognizing its binding targets^22^. Hangauer *et al*. suggested that water molecules could strengthen the cooperativity between the functional groups of the ligand in the thermolysin-binding pocket^23^. Three interfacial water molecules were identified by Christensen and coworkers as engaged in an extensive hydrogen bonding network between thapsigargin and the backbone of sarco/endoplasmic reticulum calcium ATPase (SERCA), which could influence the orientation of the thapsigargin molecule to bind to the active site^24^. In addition, Woods *et al*. addressed the impact of a conserved water molecule that mediates saccharide binding to legume lectin concanavalin A^25^. These studies have demonstrated that certain structural water molecules within protein active sites are relevant for ligand-protein recognition and contribute to binding affinity by modifying the active site geometry and generating water-mediated hydrogen bond networks, and thus should be considered in drug design^18^,^21^,^26^,^27^,^28^.

In this study, unexpected water-mediated hydrogen bond networks were identified in the interface between *h*DHODH and benzylidenehydrazinyl-substituted thiazole inhibitors in the analysis of the X-ray crystal structure of *h*DHODH in complex with the lead compound **7** (PDB ID: 4LS0), which was identified via virtual screening (Fig. 1)^6^. Three rounds of structure-guided lead optimization and X-ray crystallographic investigations revealed that the water-mediated binding modes were specific to inhibitors harboring benzylidenehydrazinyl-substituted thiazole skeletons. Compared to other published structures of *h*DHODH inhibitors, the benzylidenehydrazinyl-substituted thiazole analogs exhibited greater flexibility within the binding site due to the two rotatable bonds of the amphipathic methylenehydrazine linker connecting the hydrophilic and hydrophobic portions of the ligands. Water molecules located at the P3 and P4 positions acted as anchors to help maintaining the bioactive conformation of the inhibitors in this series. Therefore, the benzylidenehydrazinyl-substituted thiazole inhibitors developed in this study exhibited relatively high binding affinities against *h*DHODH. Of these inhibitors, compounds **19** and **30** were the most potent with the IC_50_ values in the double-digit nanomolar scale. Moreover, **19** exhibited remarkable anti-arthritic efficacy *in vivo* and markedly alleviated foot swelling in a dose-dependent manner. Compound **19** also displayed good pharmacokinetic profiles and a half-life of 9.69 h indicating its promise as a scaffold for further development as an anti-arthritic agent. Interrogating the distributions and binding patterns of all the bridging water molecules in the 35 X-ray crystal structures of *h*DHODH deposited in the Protein Data Bank^29^ suggested that the locations and binding modes of the binding-enhancing interfacial water molecules in the ubiquinone-binding site tend to be complementary with the polar moiety of the ligands and should be exploited rationally in the future drug design targeting *h*DHODH enzyme. In addition to providing novel chemical scaffolds for the treatment of rheumatoid arthritis, the present study also sheds light on the importance of water-mediated hydrogen bond networks for molecular design and optimization.

## Results and Discussion

### Lead Generation and X-ray Structure Analysis

**Figure 2:**
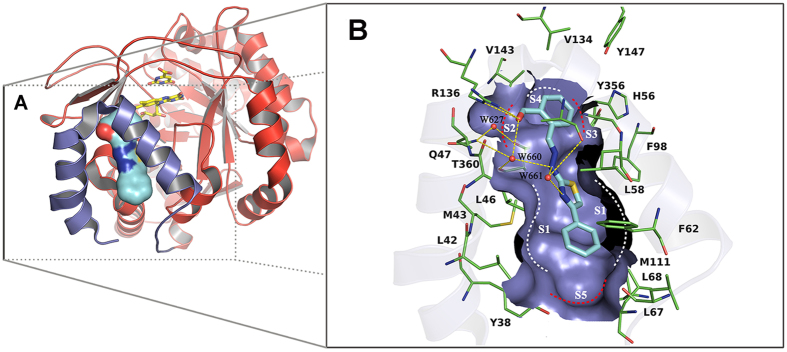
X-ray structure determination of *h*DHODH in complex with 7 (PDB ID: 4LS0). (**A**) Overview of the structure of *h*DHODH in complex with compound **7.** The receptor is shown in cartoon with the large C-terminal domain colored red and the small N-terminal domain colored purple. Flavin mononucleotide (FMN) and orotate (ORO) are displayed as yellow sticks. Compound **7** is rendered as cyan surface. (**B**) Detailed description of the ubiquinone-binding site. The hydrophilic pockets are highlighted with red dashed lines, and the hydrophobic ones are marked with white dashed lines. Critical residues are represented as thin green sticks. Oxygen atoms are colored red and nitrogen atoms blue. Water molecules are depicted as red balls. Hydrogen bonds are shown as yellow dashed lines.

Compound **7**, a benzylidenehydrazinyl-substituted thiazole derivative, was first identified in a hierarchical structure-based virtual screen by our group as a potent *h*DHODH inhibitors with an IC_50_ value of 0.365 μM^6^. To elucidate the binding mode of **7** and promote the structure-based development of *h*DHODH inhibitors of this series, compound **7** was co-crystallized with *h*DHODH, yielding X-ray structures that diffracted to 2.07 Å. The coordinates of the crystal structure have been deposited in the Protein Data Bank with PDB ID code 4LS0. The X-ray structure of **7** and *h*DHODH reveals that the formyl-substituted phenyl ring moiety of **7** is located at the inner region of the ubiquinone-binding pocket of *h*DHODH. A hydrogen bond is observed between the oxygen atom of the aldehyde group of **7** and the guanidine group of Arg136 at subsite S2 (Fig. 2). Two buried water molecules (W627 and W660) in this hydrophilic region form a complicated water-bridging hydrogen bond network with the ligand and residues Gln47, Arg136 and Thr360. More specifically, W627 forms hydrogen bonds with the sidechain nitrogen atom of Gln47 and the sidechain oxygen atom of Thr360, while W660 is more versatile and participates in the formation of four hydrogen bonds with the sidechain nitrogen atom of Gln47, the backbone oxygen atom of Thr360, the oxygen atom of the formyl group of **7**, and the secondary amine nitrogen atom of the hydrazine group of **7**. Another structural water molecule, W661, is located near the thiazole moiety and forms a bridge between the nitrogen atom of the thiazole group, the secondary amine of the hydrazine moiety and the carbonyl oxygen atom of residue Ala55. Thus, W661 not only enhances the intramolecular interactions within **7**, but also participates in polar interactions between the ligand and *h*DHODH within the hydrophobic subsite S1. In addition to the hydrogen bond interactions in the hydrophilic region, the hydrophobic interactions between **7** and *h*DHODH near the entrance of the ubiquinone-binding site (S1, Fig. 2) are also of crucial importance. Because the 4-phenyl-thiazole moiety extends to the entrance of the pocket and fits the pocket shape well, hydrophobic effects and VDW interactions occur between the 4-phenyl-thiazol moiety and residues Leu42, Met43, Leu46, Leu58, Phe62, Leu68, Phe98 and Met111.

Analysis of the binding mode of the lead compound in the X-ray structure highlighted three water molecules (W627, W660 and W661) embedded in the binding site. Compared with the water molecule W627, which consistently bridges Gln47 and Thr360 of the receptor and the polar groups (like carbonyl, carboxyl and cyano) of the ligands in subsite S2 (Fig. 2), W660 and W661 exhibit unexpected binding modes by engaging the 2-(2-methylenehydrazinyl)thiazole moiety of **7** in close contacts with the receptor. The methylenehydrazine linker that connects the hydrophilic and hydrophobic portions of the inhibitor provides two rotatable bonds and results in a more flexible structure. Therefore, the presences of W660 and W661 act as anchors to maintain the skeleton of **7** in its bioactive conformation, thereby enhancing the binding affinity between **7** and *h*DHODH.

To identify more potent *h*DHODH inhibitors and to determine if the three water molecules, particularly W660 and W661, are present in other X-ray structures of *h*DHODH in complex with the benzylidenehydrazinyl-substituted thiazole analogs, lead optimization was conducted in three phases: i) a substructure-based database search to diversify the scaffold of the lead compound and obtain beneficial information; ii) hydrophilic modification of the benzene ring located at subsite S2 to improve polar contacts and determine if variation of the hydrophilic substitutions will influence the presence of the buried water molecule located within the binding site; and iii) hydrophobic optimization at subsite S1 to strengthen nonpolar contacts and to determine if the appearance of the structural water molecule is affected by stronger hydrophobic interactions.

### First-Round Optimization of *h*DHODH Inhibitors

To detect what functional groups are more favorable for the binding at subsite S2, the substructure-based database searching approach was applied in the first round against the currently available databases (such as SPECS and MayBridge database) to rapidly obtain the derivatives. Using unsubstituted (*E*)-2-(2-benzylidenehydrazinyl)-4-phenylthiazole as the query scaffold, a focused library containing several thiazole derivatives was extracted from the SPECS database^30^, and all compounds in this focused library (**8**–**14**) were purchased from the vendor. HPLC analysis data confirmed the purity of these compounds to be ≥95% (Supplementary Table S1). However, none of the analogs were active (Table 1). Combined with the binding information of **7**, we then analyzed the structure-activity relationship of compounds **8**–**14** and synthesized **15**–**18**. Compounds **15** and **18** exhibited moderate inhibition of *h*DHODH with increased lipophilicity, resulting in sharply decreased ligand lipophilicity efficiency (LLE)^31^,^32^. In contrast to the formyl group of **7**, compounds **8**–**18** contained a hydroxyl-substituted or unsubstituted aromatic ring in subsite S2 (Fig. 2), which undermined the hydrogen bond interactions between the compounds and residues Arg136 and Gln47. This led to a sharp decrease in inhibitory activity as well as a disfavored increase in ligand lipophilicity. By comparing the inhibitory activity of the compounds in the first-round, it was indicated from the opposite side that a carbonyl-substituted aromatic ring in R^2^ is more preferred than the hydroxyl-substituted or unsubstituted one by subsite S2. In addition, an *o*-chlorine atom in the benzene group of the 4-phenyl-thiazol moiety was tolerated (**18**). Accordingly, in the next round of optimization, the accepted *o*-chlorine atom located in hydrophobic subsite S1 was retained and more polar group was introduced at subsite S2.

### Second-Round Optimization of *h*DHODH Inhibitors

Based on results of the first round, a carboxyl group was introduced into the phenyl to replace the hydroxy group. The resulting compound **19** had an IC_50_ value of 0.032 μM against *h*DHODH, a 43-fold increase compared to the hydroxy-substituted derivative **18**. Coupled with a reduction in lipophilicity of more than one log unit lipophilicity, **19** exhibits a pronounced advantage over **18** in terms of LLE. Obviously, the greatly increased activity of **19** is attributable to the strong salt bridge interaction between the carboxyl group and Arg365. However, due to an inappropriate orientation and steric clashes, compounds with a meta-substitution (**20**) or para-substitution (**21**) of the carboxyl group were unable to directly interact with Arg136, thus leading to a loss of activity. Esterification of the ortho-substituted carboxyl group of **19** with methyl alcohol (**22**) or ethyl alcohol (**23**) affected the aforementioned salt bridge, resulting in decreases in potency of 101- and 58-fold, respectively, compared with **19**. The esterification also increased the lipophilicity of **22** and **23** compared to **19,** leading to an obvious decrease in LLE.

Compound **23** was slightly more potent than **22**, which may be attributable to the stronger hydrophobic interactions between the ethyl group and the small hydrophobic pocket formed by residues Val134 and Val143 at subsite S4 (Fig. 2). To detect subsite S4, a fluorine atom was introduced (**24**). Unfortunately, **24** displayed a 7-fold decrease in inhibition activity compared with **19**. This decrease is attributable to the effect of the electron-withdrawing nature of the fluorine atom impacting the orientations of the two oxygen atoms of the carboxyl group, which slightly weakens the salt bridge and the water-mediated hydrogen bond interaction network. In an attempt to establish polar contacts with residues Tyr356, His56 and Tyr147, an amino group was introduced at subsite S3 (**25**). **25** exhibited a sharp decrease in inhibitory activity, which was likely due to steric clashes. In summary, the second round of optimization revealed that the derivative with an ortho carboxyl-substituted group on the benzene ring at subsite S2 was strongly preferred.

### Analysis of the X-ray Structure of 19 with *h*DHODH.

**Figure 3:**
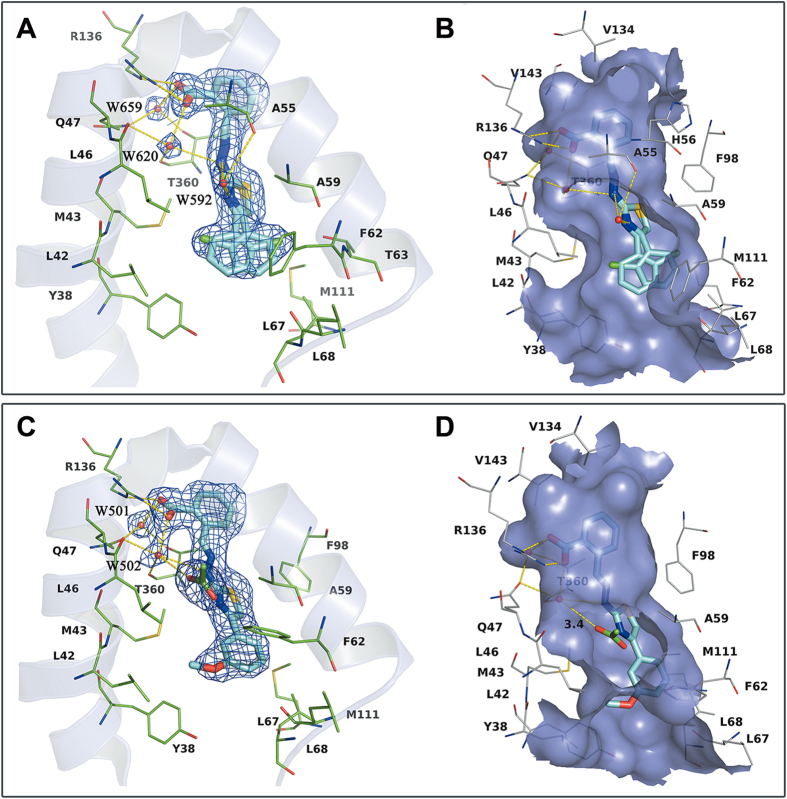
X-ray structure determination of *h*DHODH in complex with 19 (PDB ID: 4LS1) and 27 (PDB ID: 4LS2). (**A**) 2*Fo*-*Fc* electron density (blue) for **19** contoured at 1σ. Critical residues are represented as thin green sticks. (**B**) Specific binding information of the tunnel-like binding site of 4LS1. The surface of the binding site is colored purple. Compound **19** is displayed as cyan sticks and important residues are represented as gray lines. (**C**) 2*Fo*-*Fc* electron density (blue) for **27** contoured at 1σ. (**D**) Specific binding information of the tunnel-like binding site of 4LS2. Compound **27** (cyan) and acetate molecule (green) are displayed in stick mode. Water molecules are displayed as red balls and hydrogen bonds are shown as yellow dashed lines.

Using our established X-ray crystallographic protocols (see Experimental Section), we obtained a 2.20 Å X-ray structure of compound **19** in complex with *h*DHODH. The coordinates of the complex structure have been deposited in the Protein Data Bank as the entry 4LS1. The binding mode of compound **19** is presented in Fig. 3A,B. A similar water-bridging hydrogen bond network was observed around the benzylidenehydrazinyl-substituted thiazole portion of inhibitor **19**. Crystallographic water molecules (W592, W620, and W659) were observed at nearly the same location and function in the same way as those depicted in Fig. 2. W659 mediates the interaction between Gln47, Thr360 and the carboxyl group. W620 is also tetra-coordinated with the ligand and the protein by simultaneously forming two hydrogen bonds with the receptor and another two with compound **19**. A 1.1-Å deviation is apparent between W592 and W661 in Fig. 2. Compared to W661, W592 is closer to Ala55 and slightly further from the hydrazine moiety. However, the bridging functions of W592 are unaffected, and W592 is involved in three hydrogen bonds with the nitrogen atom of the thiazole group, the secondary amine of the hydrazine moiety and the carbonyl oxygen atom of residue Ala55. The novel binding modes of W592 and W620, coupled with the typical binding pattern of W659, result in the formation of a complicated hydrogen bond network that contributes to the stability of the bioactive conformation of the flexible scaffold provided by the benzylidenehydrazinyl-substituted thiazole moiety, which enables favorable polar interactions with *h*DHODH. Moreover, in subsite S2 of the ubiquinone-binding site, the carboxyl of the benzoic acid moiety forms a nearly perfectly oriented salt bridge with the side chain of Arg136. The combination of this favorable salt bridge and the strong water-bridging hydrogen bond network increase the potency of compound **19** compared to compound **18**.

Notably, the chlorophenyl group exhibits a dual binding mode at the entrance of the binding tunnel. The chlorophenyl moiety perfectly accommodates the concave surfaces formed by Met43 and Leu46 on the left side and Ala59, Phe62, and Met111 on the right side (Fig. 3B). In addition to the shape complementarity, the *o*-substitution of a chlorine atom on the benzene group presumably has the same favorable effects on binding affinity as the methyl group by stabilizing the bioactive conformation of compound **19**. This conformation more closely matches the conformer observed in the X-ray structure of the *h*DHODH and **19**, thus decreasing the conformational reordering required upon binding^33^,^34^,^35^. The amphipathic character, salt bridge, water mediation effect and conformational advantages combine to establish compound **19** as the most potent *h*DHODH inhibitor among the benzylidenehydrazinyl-substituted thiazole derivatives, with an IC_50_ of 32 nM.

### Third-Round Optimization of *h*DHODH Inhibitors

A third round of modification was launched to further determine if the water-bridging hydrogen bond network would be destroyed when the R^4^ group (2-chlorine-Ph) of **19** was replaced by more diverse substituents. When there was no substitution on the phenyl moiety, the hydrophobic interactions were weakened, resulting in a slight decrease in activity (**26**). When another chlorine atom was introduced at the 5-position of the phenyl moiety, compound **30** (IC_50_ = 0.043 μM) did not exhibit any advantage over **19**. Because **30** exhibited increased lipophilicity but similar potency, resulting in a decreased LLE value. Baumgartner *et al*.^11^ proposed a new, predominantly polar subsite S5 (Fig. 2) consisting of the hydroxy group of Tyr38 and the backbone carbonyl of Leu67 in the hydrophobic section. This subsite was probed by attaching a -OCH_3_ group to the 3-position of the phenyl moiety (**27**). Unfortunately, no obvious polar interactions were observed between the proposed subsite S5 and the -OCH_3_ moiety (Fig. 3C). However, **27** was equipotent with **19** in terms of LLE, as its reduced lipophilicity compensated for its decreased potency. Efforts to explore this subsite were continued, and **28** was synthesized. The lipophilicity of **28** was reduced by two log units at the expense of a sharp decrease in potency, making **28** less attractive than **19**. Therefore, attempts to generate polar contacts with residues Tyr38 and Leu67 at subsite S5 by modifying the 3-position of the phenyl group were unsatisfactory. In addition, the binding mode of **27** in the crystal structure (Fig. 3C) also suggested that substitutions at the 4-position of the phenyl moiety could be accommodated. The biological activity data for **29** and **32** indicated that small hydrophobic groups such as -CH_3_ are favored at the 4-position, whereas larger groups such as phenyl, may collide with Leu67 and Leu68 and result in a loss of activity. Interestingly, when the biphenyl group was converted into a naphthalene group (**31)**, the latter was capable of forming π-π stacking interactions with Phe62. Thus, compound **31** exhibited moderate inhibitory activity. None of the compounds in the third round performed better than **19**, but this round revealed that small substituting groups such as -Cl, -CH_3_ and -OCH_3_ are favored at the entrance of the binding site, whereas substituents at the 4-position of the phenyl moiety are actually size-constrained. More importantly, the crystal structure of *h*DHODH in complex with **27** was solved (PDB ID: 4LS2), which confirmed the functions of the versatile water molecules observed in the other structures.

### Analysis of the X-ray Structure of 27 with *h*DHODH

The X-ray structure of *h*DHODH with compound **27** was obtained, and the binding mode of **27** is shown in Fig. 3C,D. Interestingly, there are only two structural water molecules (W501 and W502) in the inner polar site of the tunnel-like binding site. These water molecules form nearly the same water-bridging hydrogen bond network observed in compounds **7** (W627 and W660) and **19** (W659 and W620). W501 mediates the interaction between Gln47, Thr360, and the polar carboxyl group of **27**. The versatile tetra-coordinated W502 appears in the same place and mediates interactions in the same manner as W660 and W620, even though some modifications to the benzene motif stretching along the hydrophobic entrance of the binding site are apparent. Nevertheless, an acetate molecule instead of a water molecule (W661 in Fig. 2, W592 in Fig. 3A) is in close proximity to the thiazole moiety of the inhibitor, which came from the crystallization agent. The size of the final 2*Fo-Fc* electron density map at this site is too big for one water molecule but most suitable for one acetate molecule, and the acetate molecule happened to locate at the entrance of the inhibitor binding pocket and formed hydrogen bonds with compound **27** in the complex structure. In addition to the polar contacts with the nitrogen atom of the thiazole group and the secondary amine of the hydrazine moiety, this polar solvent molecule also forms hydrogen bonds with W502 at a distance of 3.4 Å. This structure indicates that the water molecules positioned at W501 and W502 (Fig. 3C) are stable and complement the binding of the scaffold of the benzylidenehydrazinyl-substituted thiazole inhibitor and *h*DHODH. However, the water molecule located around the thiazole portion of the ligand is loosely bound in the *h*DHODH-ligand system and is susceptible to be replaced by a solvent molecule. As shown in Fig. 3B,D, the hydrophobic entrance of the binding pocket is exposed to solvent, and its openness is largely influenced by the rotation of the flexible residue Phe62.

In addition to the water-bridging interactions, the salt bridge between residue Arg136 and the carboxyl moiety of **27** is also a valuable feature for binding affinity. The -OCH_3_ group at the 3-position of the phenyl moiety matches the concave surface perfectly, forming close hydrophobic interactions with the residues of Leu42, Met43 and Leu46 in subsite S1. Figure 3C also indicates that little space remains between the 4-position of the phenyl moiety and the receptor at the entrance of the binding site, and only small substitutions can be accommodated, as verified using compounds **29** and **32**. This round of X-ray crystallographic exploration confirmed once again that the binding of the benzylidenehydrazinyl-substituted thiazole inhibitor to *h*DHODH is accompanied by two tightly bound water molecules and a loosely bound water molecule that is exposed to solvent.

### Analysis of Structural Water Molecules in the Ubiquinone-Binding Site.

**Figure 4:**
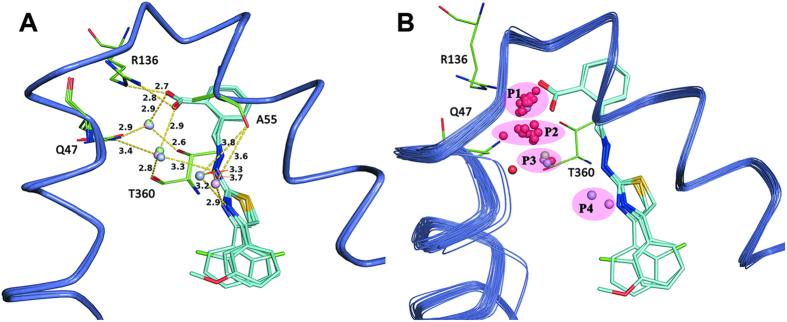
Analysis of the structural water molecules at the ubiquinone-binding site of *h*DHODH. (**A**) Water-bridging hydrogen bond network. Three X-ray crystal structures solved in this study are aligned together (purple ribbon) and the corresponding ligand are displayed as thin cyan sticks. Interfacial water molecules of 4LS0 (lightblue), 4LS1 (lightpink) and 4LS2 (palegreen) are shown as round balls. Hydrogen bonds are rendered as yellow dashed lines. (**B**) Distributions of the buried water molecules. Besides the three structures, all the other 32 crystal structures of *h*DHODH deposited in the Protein Data Bank previously are aligned together, and their buried water molecules are colored red.

In each of the crystal structures of the three complexes derived in our study, an unexpected water bridging pattern was discovered (Fig. 4A). Figure 4B presents the alignment of all 32 reported protein-ligand complex structures of *h*DHODH deposited in the Protein Data Bank (Supplementary Information Table S2), with the addition of the 3 structures solved in this study. In total, there are 43 interfacial water molecules clustered into four positions in the ubiquinone-binding site of these 35 crystal structures, of which 58.1% are located in the P1 position and 23.3% are located in the P2 position. Of the five water molecules at the P3 position, three were identified in this study. In addition, two water molecules were observed at the P4 position for the first time in this study.

Apparently, more than half of the interfacial water molecules are located at the P1 position, where they perform the general functions of bridging Gln47, Thr360 and polar groups (like carbonyl, carboxyl and cyano) of the ligands. Therefore, the water molecule at the common P1 position is adequately considered in molecular docking or virtual screening work^6^. The appearance of a water molecule at the P2 position is always accompanied by polar groups such as that of A771726. In detail, the water molecule at the P2 position is involved in an intricate hydrogen bond network between residues Gln47, Arg136 and the carbonyl group of the amide moiety in the A771726-like ligand. This binding mode was observed in several X-ray structures, such as 1D3H, 3F1Q, 3FJL, 3G0U, 3G0X and 3U2O. By contrast, unlike the water molecule commonly observed near the P1 or P2 position, the water molecules located at the P3 and P4 positions were rarely observed in other previously published X-ray structures of *h*DHODH. Interestingly, the co-occurrences of water molecules at the P3 and P4 positions were found together with the scaffold of the benzylidenehydrazinyl-substituted thiazole inhibitors designed in this study. These water molecules cooperate with each other to form a complicated hydrogen bond network that facilitates the binding of the inhibitors in this series to the active site of *h*DHODH. The water molecule at P3 region was tetra-coordinated with the ligand and the protein through an unexpected bridging pattern in which the water molecule simultaneously forms two hydrogen bonds with the ligand (the benzylidenehydrazinyl portion) and an additional two hydrogen bonds with the protein (the residues Gln47 and Thr360). Moreover, three rounds of verification demonstrated that the water molecule at the P3 position is robust, tightly bound and not shifted by molecular decoration at the hydrophilic or hydrophobic portions of lead compound **7**. The water molecule at P3 position appears to be a specialized binding partner that maintains the bioactive conformation of the benzylidenehydrazinyl-substituted thiazole inhibitor, which has a more flexible methylenehydrazine linker connecting the polar and nonpolar portions of the ligand in the binding site compared to other known inhibitors. The water molecule located at the P4 position can also bridge the nitrogen atom of the thiazole group, the secondary amine of the hydrazine moiety, and the carbonyl oxygen atom of residue Ala55. Thus, this water molecule enhances intramolecular interactions in addition to participating in polar interactions between the ligand and *h*DHODH in the hydrophobic subsite S1. Therefore, the water molecules at the P3 and P4 positions actively contribute to maintaining the bioactive conformations of the benzylidenehydrazinyl-substituted thiazole inhibitors in the ubiquinone-binding site of *h*DHODH. However, the water molecule at the P4 position is bound more loosely than the water molecule located at the P3 Position, and thus is susceptible to be replaced by the polar acetate molecule (Fig. 4) because the P4 position is exposed to solvent.

Analysis of the distributions and binding modes of the water molecules in the ubiquinone-binding site of *h*DHODH indicated that the hydrophilic pocket of the active site is water accessible and that the locations and binding patterns of the water molecules complement the polar moiety of the ligands that bind to *h*DHODH. Given the crucial role of water in protein-ligand association, water molecules should be exploited in the design of *h*DHODH inhibitors.

### *In vivo* Pharmacokinetic Study of Compound 19

A pharmacokinetic study of the most potent inhibitor, **19**, was performed by administering rats a 1 mg/kg intravenous (IV) dose and a 10 mg/kg oral dose (PO) of the compound. After IV dosing, **19** exhibited a terminal half-life of 9.69 h, a steady-state volume of distribution of 0.35 L/kg, and a low plasma clearance of 0.04 L/h/kg (Table 2). After oral administration, **19** exhibited an exposure (AUC_0-∞_) of 53047.73 μg/L*h, leading to an oral bioavailability of 22.75%. The maximum plasma concentration (C_max_) of **19** was 5310.50 μg/L, and the time to reach the maximum concentration (*T*_max_) of the inhibitor was 1.00 h. Compared with Vidofludimus (4SC-101), which targets *h*DHODH and is in phase II clinical trials for the treatment of rheumatoid arthritis and inflammatory bowel disease (with a terminal half-life of 2.3 to 3 hours in MRL^lpr/lpr^ mice^36^,^37^), **19** likely has an advantage in terms of its terminal half-life (9.69 h). The oral bioavailability of **19** could be improved by administration in its salt formation and their drug-like properties along with pharmacokinetic characteristics will be optimized further in the future work.

### *In vivo* Anti-arthritic Efficacy of Compound 19.

**Figure 5:**
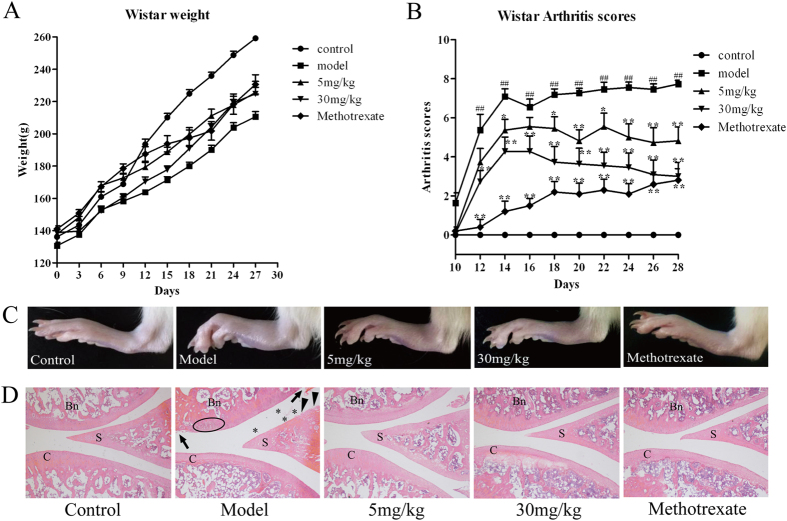
*In vivo* effects of compound **19** on collagen-induced arthritis (CIA) rats. Arthritis was induced in Wistar rats by twice immunization with type II collagen on day 0 and day 7. Compound **19** was administered i.p. daily at 5 mg/kg and 30 mg/kg until the end of this experiment. Methotrexate, as the positive control, was administered at the dose of 0.3 mg/kg. (**A**) Measurement of body weight was taken every three days. Treatment of compound **19** or methotrexate had no obvious effect on the growth of body weight versus model group. (**B**) The incidence and severity of arthritis in different groups were evaluated via observation of the changes of arthritis scores every two days. Compound **19** displayed significant anti-arthritic effect *in vivo* (p < 0.05) and obviously alleviated foot swelling in a dose-dependent manner. (**C**) The representative photomicrographs of knee joints in different treatments groups taken after 28 day’s trial. The arthritis swelling of the model group was the worst. The swelling hind paw was obviously relieved in the group treated with compound **19**. (**D**) Representative H & E stained sections of joint tissue of each groups. Compared with model group and compound **19** treated groups, the latter could remarkably alleviate the histological changes of severe arthritis. Asterisk indicates the infiltration of the inflammatory cells; solid triangles indicate areas with synovial degradation; solid arrows indicate areas of cartilage degradation; and the oval highlights the inflammatory cells. S, synovium; C, cartilage; Bn, bone. Magnification = 50 ×. Data was expressed as mean ± SEM. ^##^P < 0.01 *vs* normal control, *P < 0.05, **P < 0.01 *vs* CIA group.

Compound **19** and methotrexate were injected intraperitoneally once per day for 28 days into the Wistar rats with collagen-induced arthritis (CIA). The swelling scores of the arthritis and morphological observations of the rats’ joint tissues were employed to examine the anti-arthritic effect of compound **19** (Fig. 5). With the onset of arthritis, the swelling scores were an indication of the disease states. The maximum score was 8, which is the sum of the scores from both hind paws of each rat and indicates a severe arthritis state. During the experiment, the control rats had normal eating patterns, shiny hair and continuously increasing body weight. By contrast, the rats in the model group exhibited a rough and dull hair at the beginning of the trial, and a slower increase in body weight relative to the normal group after day 9 (Fig. 5A). Although treatment with compound **19** or methotrexate had no obvious influence on increase in body weight, compound **19** displayed significant dose-dependent anti-arthritic effect (p < 0.05) and obviously alleviated foot swelling (Fig. 5B,C). The arthritis scores of the CIA rats reached their peak and stabilized (Fig. 5B) around 8 after day 18, presenting a severe arthritis state, while the arthritis scores of the compound **19** treated rats decreased and were around 5 (5 mg/kg) and 3 (30 mg/kg) at the day 28, which indicated that compound **19** can display substantial anti-inflammation effects and alleviate foot swelling in a dose-dependent manner (Fig. 5B,C).

Besides, histological changes in the joint tissues of the normal, CIA, compound **19** and methotrexate treated rats were investigated by the hematoxylin and eosin (H&E) staining method. In the joint tissues of the CIA rats with serious arthritis (Fig. 5D), pathological features including the infiltration of the inflammatory cells (marked with asterisk), synovial degradation (marked with solid triangles), cartilage erosion in the joint tissues (marked with solid arrows) and inflammatory lesions of some cells (highlighted with an oval) were clearly observed. Besides, due to the serious arthritis, the articular cavity was full of exudate which was larger than the other compound treated groups. Compared to the CIA rats, the previously mentioned pathological changes were evidently alleviated in the joint tissues of the rats treated with compound **19** in both dosage, demonstrating that compound **19** displayed anti-arthritic effects in a dose-related manner. The significant *in vivo* anti-arthritic effects of **19** not only confirmed that *h*DHODH is an effective anti-arthritic target but also confirmed that this compound is a promising chemical scaffold for further development as an immunosuppressant and antiproliferative agent targeting *h*DHODH.

## Conclusion

In summary, this work presented a series of benzylidenehydrazinyl-substituted thiazole inhibitors to suppress the bioactivity of *h*DHODH. Compound **19** displayed the lowest IC_50_ value among these compounds, with notable anti-arthritic efficacy and acceptable pharmacokinetic profiles *in vivo*. These results suggest the potential of **19** for application as an immunosuppressive agent targeting *h*DHODH. More importantly, water-bridged interactions between the ligands and *h*DHODH were identified in the three X-ray structures solved in this study. Two robust bridging water molecules co-occurred with the ligands to help maintain their bioactive conformations by generating a strong water-mediated hydrogen bond network between the flexible 2-(2-methylenehydrazinyl)thiazole linker and *h*DHODH. The locations and H-bonding patterns of the water molecules are highly complementary with the polar moieties of the ligands in the ubiquinone-binding site of *h*DHODH and should be considered in future drug design and optimization workflows targeting *h*DHODH.

## Methods

### *In vitro* Enzyme Assay

The plasmid coding for human DHODH was kindly provided by Prof. Jon Clardy (Harvard Medical School)^7^. *h*DHODH (Met30-Arg396) was expressed and purified in the same manner as described in our recently published work^38^. We determined the effect of benzylidenehydrazinyl-substituted thiazole derivatives to inhibit *h*DHODH by using a DCIP assay method^38^. The assay buffer contained 50 mM HEPES pH 8.0, 150 mM KCl, 0.1% Triton X-100, 100 μM UQ_0_ and 120 μM DCIP. The results were presented as the mean ± standard deviation of three independent experiments (Table 1).

### Crystal Structure Determination

Co-crystallization of *h*DHODH with compound was performed as previously described^38^. X-ray diffraction data were collected at 100 K at the synchrotron beamline BL17U1 at SSRF (Shanghai, China). Statistics of data collection, processing, and refinement were summarized in Table 3. The data were processed with MOSFLM^39^, and scaled using the SCALA program from the CCP4 suite^40^. Structural elucidation and refinement were carried out using the CCP4 suite of programs^40^,^41^. The PDB entry 1D3G (stripped of ligands and water molecules) was used as a molecular replacement model for phasing of the X-ray data. Iterative model building and refinement were performed using Coot and Refmac5, respectively^42^,^43^. The molecular graphics package PyMOL (DeLano, 2002) was used to generate the figures.

### *In vivo* Pharmacokinetic Assay

Compound **19**, the most potent inhibitor, was selected to perform the single dose pharmacokinetic studies. Male Sprague-Dawley rats were raised according to the Guide for the Care and Use of Laboratory Animals. Blood samples of the rats were collected at the intervals of 5 min, 15 min, 30 min, 1 h, 2 h, 4 h, 8 h and 24 h. Heparin sodium was applied as the anticoagulant of the blood samples. Based on the LC/MS/MS quantitative data, pharmacokinetic parameters were analyzed using WinNonlin5.2 with noncompartment model.

### *In vivo* Efficacy Study

Adult male Wistar rats (120–140 g) were purchased from Shanghai SLRC Laboratory animal company and housed in standard pathogen-free cages. The environment was climate-controlled (20–25 °C, 50–60% relative humidity) with a 12-hour light and 12-hour dark cycle as described previously^38^. Sterilized food and water were provided naturally. All procedures involving animals conform to the Chinese government guidelines for animal experiments, and were conducted with approval of the Institutional Animal Care and Use Committee of East China University of Science & Technology. Bovine type II collagen (CII, Chondrex, USA) was emulsified in the same manner as that conducted previously to get the CIA model^44^. Specifically, 49 rats were randomly assigned into five groups: normal control (solvent, i.p., n = 6), CIA model (solvent, i.p., n = 11), Methotrexate-treated (0.3 mg/kg, i.p., n = 10), compound **19**-treated (5 mg/kg, i.p., n = 11) and compound **19**-treated (30 mg/kg, i.p., n = 11) groups. Methotrexate, reported to has a therapeutic effect on rheumatoid arthritis^45^, was used as the positive control in this study. The main workflows of this experiment were consistent with that reported recently^38^. During the 28 days of the experiment, compound **19** (5 mg/kg and 30 mg/kg) and Methotrexate (0.3 mg/kg) were injected intraperitoneally once every day. At the same time, the normal control group and model group were administered the same amount of solution. The solvent of **19** was made up of 5% DMSO, 45% saline and 50% PEG400.

In this study, the clinical-mimic arthritis was observed and the severity was assessed by the same criteria as that applied in reference 65: 0, normal, without any swelling; 1, mild swelling; 2, swelling; 3, significant swelling; 4, severe swelling. The maximum score was 8, which was the sum of the scores from both hind paws of each rat. When arthritic signs appeared, the incidence and severity of the arthritis in rats with different treatments were evaluated via arthritis scores recorded every 2 days under blinded conditions. Measurement of body weight was taken every 3 days. All Wistar rats were killed after 28 day’s trails. Pathological detections against the whole knee joints of the rats were conducted using the same method as described previously^38^. Mean ± standard deviation (mean ± SEM) was applied to present the data in this study. SPSS was used to evaluate the statistical significance of differences among the groups. P ≤ 0.05 was the threshold to be statistically significant^46^.

### Chemistry

A series of benzylidenehydrazinyl-substituted thiazole derivatives were designed and synthesized as potential *h*DHODH inhibitors. These analogs were primarily prepared via the cyclization of thiosemicarbazone with 2-bromo-1-phenylethanones^47^, and the thiosemicarbazones were obtained from the condensation reaction of commercially available substituted benzaldehydes and thiosemicarbazide^47^. The preparation of 2-bromo-1-phenylethanones began with commercially available acetophenones with diverse substituents^48^. Following this design route, we successfully acquired compounds **15–18** and **20–21** (as shown in Supplementary Scheme S1). When the substituted benzaldehyde was transformed to the *o*-carboxybenzaldehyde, further intramolecular cyclization between the carboxyl group and the second nitrogen atom in the hydrazine group occurred in addition to the anticipated reaction (as shown in Supplementary Scheme S2). To obtain the target compounds, extensive efforts were made to regulate the hydrolysis of the intramolecular amide bond. Fortuitously, this problem was resolved by referring to an article in which NaOH was used in dry THF^49^. However, several substituted benzaldehydes, such as **24a** and **25a** in Supplementary Scheme S2, were commercially unavailable. Therefore, a five-step workflow comprising esterification, benzylic bromination, lactonization, benzylic bromination and hydrolysis was designed to obtain their crucial *o*-carboxybenzaldehyde intermediates (**24V** and **25V**). For this workflow, 2-methyl benzoic acid derivatives were used as the starting products (Supplementary Scheme S3)^50^,^51^,^52^,^53^,^54^,^55^,^56^,^57^.

The preparations of the esterified derivatives (**22**, **23**) are shown in Supplementary Scheme S4. For these compounds, the esterification reactions of 2-formylbenzoic acid were firstly performed to obtain the methyl ester and ethyl esters without affecting the aldehyde group^58^. Thiazole hydrazine was obtained via the condensation of thiosemicarbazide and 2-bromo-1-phenylethanones in 1,4-dioxane at room temperature. Finally, the target products, the benzylidenehydrazinyl-substituted thiazole analogs, were obtained via a dehydration reaction of methyl or ethyl 2-formylbenzoate and thiazole hydrazine^59^. In general, all reaction conditions were mild, and no dangerous or toxic materials were used in the reactions. The structures of all products were verified by ^1^H NMR, ^13^C NMR and HRMS (ESI).

#### General methods

All chemical reagents and solvents were obtained from commercial sources and used without further purification. Thin-layer chromatography (TLC) was carried out to monitor the process of reactions. Purification of compounds was achieved by column chromatography with silica gel (HaiYang, Qingdao) 200–300 mesh. ^1^H NMR and ^13^C NMR spectra were recorded on a Bruker AM-400 spectrometer with chemical shifts expressed as ppm (in CDCl_3_ or DMSO-*d*_*6*_, Me_4_Si as internal standard). Melting points were recorded on a WRS-1B-digital melting point apparatus. The mass spectra were measured at The Institute of Fine Chemistry of ECUST. The MS data were obtained by Agilent 5975C GC-MS instrument and Agilent LC-MS 1200 and 6120 system. Analytical HPLC was performed on a Hewlett-Packard 1100 chromatography system equipped with photodiode array detector and a Zorbax XDB-C18 column (250 mm × 4.6 mm) was used to determine the purity of the products. The mobile phase A was water with 1‰ TFA and mobile phase B was acetonitrile. A gradient of 30–100% B over 20 minutes was run at a flow rate of 1.0 mL/min. Compounds synthesized in our laboratory were generally varied from 95% to 99% in purity, and the biological experiments were employed on compound whose purity is at least 95%.

#### methyl 2-(bromomethyl)-6-fluorobenzoate (24III)

^1^H NMR (400 MHz, CDCl_3_): δ 7.39 (m, 1H), 7.22 (d, *J* = 7.6 Hz, 1H), 7.09 (t, *J* = 8.4 Hz, 1H), 4.66 (s, 2H), 3.99 (s, 3H). GC-MS (EI), 246.1.

#### methyl 2-(bromomethyl)-3-nitrobenzoate (25III)

^1^H NMR (400 MHz, CDCl_3_): δ 8.12 (d, *J* = 8.0 Hz, 1H), 7.97 (d, *J* = 8.0 Hz, 1H), 7.56 (t, *J* = 8.0 Hz, 1H), 5.17 (s, 2H), 4.01 (s, 3H). GC-MS (EI), 273.0.

#### 7-fluoroisobenzofuran-1(3H)-one (24IV)

^1^H NMR (400 MHz, CDCl_3_): δ 7.69 (m, 1H), 7.29 (d, *J* = 7.6 Hz, 1H), 7.17 (t, *J* = 8.4 Hz, 1H), 5.33 (s, 2H). GC-MS (EI), 152.2. The ^1^H NMR data are matched with the data in the literature^60^.

#### 4-nitroisobenzofuran-1(3H)-one (25IV)

^1^H NMR (400 MHz, CDCl_3_): δ 8.55 (d, *J* = 8.0 Hz, 1H), 8.29 (d, *J* = 7.6 Hz, 1H), 7.83 (t, *J* = 8.0 Hz, 1H), 5.78 (s, 2H). GC-MS (EI), 179.0. The ^1^H NMR data are matched with the data in the literature^61^.

#### 2-fluoro-6-formylbenzoic acid (24V)

^1^H NMR (400 MHz, DMSO-*d6*): δ 8.27 (s, 1H), 7.84 (m, 1H), 7.50 (m, 2H), 6.66 (s, 1H). MS (ESI) m/z 167.1 [M − H]^−^.

#### 2-formyl-3-nitrobenzoic acid (25V)

^1^H NMR (400 MHz, DMSO-*d6*): δ 8.54 (d, *J* = 8.4 Hz, 1H), 8.51 (d, *J* = 8.0 Hz, 1H), 8.27 (d, *J* = 7.6 Hz, 1H), 7.97 (t, *J* = 8.0 Hz, 1H), 7.08 (d, *J* = 7.6 Hz, 1H). MS (ESI) m/z 194.1 [M − H]^−^.

#### 2-(2-hydroxybenzylidene)hydrazinecarbothioamide (15a)

^1^H NMR (400 MHz, DMSO-*d*_6_): δ 11.37 (s, 1H), 9.99 (s, 1H), 8.37 (s, 1H), 8.09 (s, 1H), 7.92 (d, *J* = 8.4 Hz, 1H), 7.91 (s, 1H), 7.21 (t, *J* = 7.6 Hz, 1H), 6.91 (d, *J* = 8.4 Hz, 1H), 6.82 (t, *J* = 7.6 Hz, 1H). MS (ESI) m/z 194.0 [M − H]^−^.

#### 2-((2-carbamothioylhydrazono)methyl)benzoic acid (19a)

^1^H NMR (400 MHz, DMSO-*d*_6_): δ 13.28 (s, 1H), 11.59 (s, 1H), 8.79 (s, 1H), 8.25 (d, *J* = 7.2 Hz, 1H), 8.22 (s, 1H), 7.96 (s, 1H), 7.83 (d, *J* = 8.0 Hz, 1H), 7.57 (t, *J* = 7.6 Hz, 1H), 7.48 (t, *J* = 7.6 Hz, 1H). MS (ESI) m/z 222.0 [M − H]^−^.

#### *3-((2-carbamothioylhydrazono)methyl)benzoic acid* (20a)

^1^H NMR (400 MHz, DMSO-*d*_6_): δ 13.11 (s, 1H), 11.48 (s, 1H), 8.23 (s, 1H), 8.21(s, 1H), 8.13 (d, *J* = 6.0 Hz, 1H), 8.12 (s, 1H), 8.09 (s, 1H), 7.95 (d, *J* = 8.0 Hz, 1H), 7.54 (t, *J* = 7.6 Hz, 1H). MS (ESI) m/z 222.0 [M − H]^−^.

#### 4-((2-carbamothioylhydrazono)methyl)benzoic acid (21a)

^1^H NMR (400 MHz, DMSO-*d*_6_): δ 13.04 (s, 1H), 11.57 (s, 1H), 8.30 (s, 1H), 8.11(s, 1H), 8.10 (s, 1H), 7.96–7.91 (m, 4H). MS (ESI) m/z 222.0 [M − H]^−^.

#### 2-((2-carbamothioylhydrazono)methyl)-6-fluorobenzoic acid (24a)

^1^H NMR (400 MHz, DMSO-*d*_6_): δ 13.86 (s, 1H), 11.68 (s, 1H), 8.40 (s, 1H), 8.20 (s, 1H), 7.93 (d, *J* = 8.0 Hz, 1H), 7.76 (s, 1H), 7.55–7.49 (m, 1H), 7.34 (t, *J* = 7.6 Hz, 1H). MS (ESI) m/z 240.0 [M − H]^−^.

#### 2-((2-carbamothioylhydrazono)methyl)-3-nitrobenzoic acid (25a)

^1^H NMR (400 MHz, DMSO-*d*_6_): δ 13.80 (s, 1H), 11.80 (s, 1H), 8.53 (s, 1H), 8.37 (s, 1H), 8.14 (d, *J* = 8.0 Hz, 1H), 8.09 (d, *J* = 8.0 Hz, 1H), 7.74 (t, *J* = 8.0 Hz, 1H), 7.08 (s, 1H). MS (ESI) m/z 267.0 [M − H]^−^.

#### (E)-2-((2-(5-methyl-4-phenylthiazol-2-yl)hydrazono)methyl)phenol (15)

mp 227.5–229.0 °C. ^1^H NMR (400 MHz, DMSO-*d*_6_): δ 11.90 (s, 1H), 10.20 (s, 1H), 8.30 (s, 1H), 7.62 (d, *J* = 7.6 Hz, 2H), 7.59 (d, *J* = 8.4 Hz, 1H), 7.44 (t, *J* = 7.6 Hz, 2H), 7.33 (t, *J* = 7.6 Hz, 1H), 7.22 (t, *J* = 8.0 Hz, 1H), 6.91 (d, *J* = 7.6 Hz, 1H), 6.88 (t, *J* = 7.6 Hz, 1H), 2.42 (s, 3H). ^13^C NMR (100 MHz, DMSO-*d*_6_): 164.2, 156.0, 140.3, 134.8, 131.5, 130.3, 128.6, 128.3, 127.9, 127.1, 126.8, 120.0, 119.4, 116.1, 12.3. HRMS (ESI) calcd for C_17_H_16_N_3_OS [M + H]^+^ 310.1014, found 310.1020. Purity: 99.51% (*t*_R_ 11.82 min).

#### (E)-2-((2-(4-(4-isopropylphenyl)thiazol-2-yl)hydrazono)methyl)phenol (16)

mp 158.0–160.0 °C. ^1^H NMR (400 MHz, DMSO-*d*_6_): δ 12.16 (s, 1H), 10.11 (s, 1H), 8.32 (s, 1H), 7.77 (d, *J* = 8.0 Hz, 2H), 7.63 (d, *J* = 7.2 Hz, 1H), 7.28 (d, *J* = 8.4 Hz, 2H), 7.23 (t, *J* = 8.0 Hz, 2H), 6.91 (d, *J* = 8.0 Hz, 1H), 6.89 (t, *J* = 7.2 Hz, 1H), 2.91 (m, 1H), 1.23 (d, *J* = 6.8 Hz, 6H). ^13^C NMR (100 MHz, DMSO-*d*_6_): 169.4, 157.3, 150.9, 149.6, 146.5, 131.9, 130.8, 130.4, 127.2, 126.7, 119.4, 117.6, 116.6, 102.2, 33.9, 23.9. HRMS (ESI) calcd for C_19_H_20_N_3_OS [M + H]^+^ 338.1327, found 338.1331. Purity: 95.76% (*t*_R_ 21.79 min).

#### (E)-2-((2-(4-(3,4-dichlorophenyl)thiazol-2-yl)hydrazono)methyl)phenol (17)

mp 226.9–228.0 °C. ^1^H NMR (400 MHz, DMSO-*d*_6_): δ 12.10 (s, 1H), 10.10 (s, 1H), 8.36 (s, 1H), 8.08 (d, *J* = 2.0 Hz, 1H), 7.84 (dd, *J*_1_ = 8.4 Hz, *J*_2_ = 2.0 Hz, 1H), 7.66 (d, *J* = 8.0 Hz, 1H), 7.64 (d, *J* = 6.8 Hz, 1H), 7.54 (s, 1H), 7.24 (t, *J* = 8.0 Hz, 1H), 6.92 (d, *J* = 8.4 Hz, 1H), 6.89 (t, *J* = 7.6 Hz, 1H). ^13^C NMR (100 MHz, DMSO-*d*_6_): 168.2, 156.0, 147.9, 134.0, 135.1, 131.4, 130.8, 130.6, 129.7, 127.2, 126.4, 125.5, 120.0, 119.5, 116.1, 105.6. HRMS (ESI) calcd for C_16_H_12_N_3_OSCl_2_ [M + H]^+^ 364.0078, found 364.0071. Purity: 99.89% (*t*_R_ 21.16 min).

#### (E)-2-((2-(4-(2-chlorophenyl)thiazol-2-yl)hydrazono)methyl)phenol (18)

mp 204.5–205.1 °C. ^1^H NMR (400 MHz, DMSO-*d*_6_): δ 12.15 (s, 1H), 10.13 (s, 1H), 8.36 (s, 1H), 7.88 (dd, *J*_1_ = 7.6 Hz, *J*_2_ = 1.6 Hz, 1H), 7.65 (d, *J* = 8.0 Hz, 1H), 7.53 (d, *J* = 7.6 Hz, 1H), 7.42 (t, *J* = 7.6 Hz, 1H), 7.36 (dd, *J*_1_ = 7.6 Hz, *J*_2_ = 1.6 Hz, 1H), 7.33 (s, 1H), 7.24 (t, *J* = 7.6Hz, 1H), 6.93 (d, *J* = 8.4 Hz, 1H), 6.90 (t, *J* = 7.6 Hz, 1H). ^13^C NMR (100 MHz, DMSO-*d*_6_): 167.1, 156.0, 147.0, 140.0, 133.2, 131.0, 130.8, 130.5, 130.3, 129.00, 127.2, 126.5, 120.8, 119.5, 116.1, 108.2. HRMS (ESI) calcd for C_16_H_13_N_3_OSCl [M + H]^+^ 330.0468, found 330.0472. Purity: 99.43% (*t*_R_ 12.44 min).

#### (E)-3-((2-(4-(2-chlorophenyl)thiazol-2-yl)hydrazono)methyl)benzoic acid (20)

mp 274.6–275.2 °C. ^1^H NMR (400 MHz, DMSO-*d*_6_): δ 12.48 (s, 1H), 8.24 (s, 1H), 8.11 (s, 1H), 7.94 (d, *J* = 8.0 Hz, 1H), 7.89 (d, *J* = 6.4 Hz, 1H), 7.87 (dd, *J*_1_ = 7.6 Hz, *J*_2_ = 1.6 Hz, 1H), 7.57 (t, *J* = 7.6 Hz, 1H), 7.54 (dd, *J*_1_ = 7.6 Hz, *J*_2_ = 1.6 Hz, 1H), 7.42 (t, *J* = 7.6 Hz, 1H), 7.36 (s, 1H), 7.35 (t, *J* = 8.0 Hz, 1H). ^13^C NMR (100 MHz, DMSO-*d*_6_): 167.7, 167.5, 147.6, 140.9, 135.3, 133.7, 131.9, 131.5, 131.2, 131.0, 130.9, 130.3, 129.7, 129.5, 127.7, 127.2, 109.3. HRMS (ESI) calcd for C_17_H_11_N_3_O_2_SCl [M − H]^−^ 356.0261, found 356.0260. Purity: 96.55% (*t*_R_ 12.89 min).

#### (E)-4-((2-(4-(2-chlorophenyl)thiazol-2-yl)hydrazono)methyl)benzoic acid (21)

mp 258.9–259.5 °C. ^1^H NMR (400 MHz, DMSO-*d*_6_): δ 12.41 (s, 1H), 8.09 (s, 1H), 7.90 (d, *J* = 8.4 Hz, 2H), 7.87 (dd, *J*_1_ = 7.6 Hz, *J*_2_ = 2.0 Hz, 1H), 7.77 (d, *J*_1_ = 8.4 Hz, 2H), 7.54 (dd, *J*_1_ = 7.6 Hz, *J*_2_ = 1.6 Hz, 1H), 7.42 (t, *J* = 7.6 Hz, 1H), 7.39 (s, 1H), 7.36 (t, *J* = 7.6 Hz, 1H). ^13^C NMR (100 MHz, DMSO-*d*_6_): 167.6, 167.4, 147.4, 140.7, 138.9, 133.6, 131.5, 131.4, 131.3, 130.9, 130.3, 129.6, 127.7, 126.7, 109.5. HRMS (ESI) calcd for C_17_H_11_N_3_O_2_SCl [M − H]^−^ 356.0261, found 356.0264. Purity: 98.38% (*t*_R_ 12.74 min).

#### 2-(4-(2-chlorophenyl)thiazol-2-yl)phthalazin-1(2H)-one (19c)

^1^H NMR (400 MHz, CDCl_3_): δ 8.83 (s, 1H), 8.44 (d, *J* = 8.0 Hz, 1H), 8.10 (t, *J* = 7.6 Hz, 2H), 8.04 (s, 1H), 8.01 (d, *J* = 8.0 Hz, 1H), 7.98 (d, *J* = 7.2 Hz, 1H), 7.60 (d, *J* = 8.0 Hz, 1H), 7.49 (t, *J* = 7.6 Hz, 1H), 7.43 (t, *J* = 7.6 Hz, 1H). ^13^C NMR (100 MHz, CDCl_3_): 158.4, 156.7, 146.8, 141.5, 135.3, 133.5, 133.3, 132.0, 131.5, 130.8, 130.1, 129.0, 128.1, 127.9, 127.0, 118.0. HRMS (ESI) calcd for C_17_H_10_N_3_OSClNa [M + Na]^+^ 362.0131, found 362.0129.

#### 2-(4-(2-chlorophenyl)thiazol-2-yl)-8-fluorophthalazin-1(2H)-one (24c)

^1^H NMR (400 MHz, CDCl_3_): δ 8.51(d, *J* = 2.4 Hz, 1H), 7.96 (dd, *J*_1_ = 7.6 Hz, *J*_2_ = 1.6 Hz, 1H), 7.93–7.87 (m, 1H), 7.73 (s, 1H), 7.63 (d, *J* = 7.6 Hz, 1H), 7.56–7.52 (m, 1H), 7.47 (dd, *J*_1_ = 8.0 Hz, *J*_2_ = 1.6 Hz, 1H), 7.35 (t, *J* = 7.6 Hz, 1H), 7.31–7.26 (m, 1H). ^13^C NMR (100 MHz, CDCl_3_): 163.1, 156.0, 155.4, 147.8, 139.7, 136.0, 133.4, 132.3, 131.8, 130.8, 130.2, 129.1, 127.0, 122.9, 120.1, 116.9, 115.9. HRMS (ESI) calcd for C_17_H_9_N_3_ONaSClF [M + Na]^+^ 380.0037, found 380.0037.

#### 2-(4-(2-chlorophenyl)thiazol-2-yl)-5-nitrophthalazin-1(2H)-one (25c)

^1^H NMR (400 MHz, DMSO-*d*_6_): δ 9.14 (s, 1H), 8.82 (d, *J* = 8.0 Hz, 1H), 8.73 (dd, *J*_1_ = 8.0 Hz, *J*_2_ = 0.8 Hz, 1H), 8.17 (t, *J* = 8.0 Hz, 1H), 8.10 (s, 1H), 7.96 (dd, *J*_1_ = 7.6 Hz, *J*_2_ = 1.6 Hz, 1H), 7.61 (dd, *J*_1_ = 8.0 Hz, *J*_2_ = 1.6 Hz, 1H), 7.49 (t, *J* = 7.6 Hz, 1H), 7.43 (t, *J* = 7.6 Hz, 1H). HRMS (ESI) calcd for C_17_H_9_N_4_O_3_NaSCl [M + Na]^+^ 406.9982, found 406.9986.

#### *2-(4-phenylthiazol-2-yl)phthalazin-1(2H)-one* (26c)

^1^H NMR (400 MHz, DMSO-*d*_6_): δ 8.85(s, 1H), 8.45 (d, *J* = 7.6 Hz, 1H), 8.11–8.01 (m, 6H), 7.49 (t, *J* = 7.6 Hz, 2H), 7.38 (t, *J* = 7.2 Hz, 1H). ^13^C NMR (100 MHz, DMSO-*d*_6_): 158.4, 157.5, 150.3, 141.4, 135.2, 134.6, 133.5, 129.2, 128.9, 128.5, 128.0, 127.0, 127.0, 126.4, 113.1. HRMS (ESI) calcd for C_17_H_11_N_3_ONaS [M + Na]^+^ 328.0521, found 328.0524.

#### *2-(4-(2-methoxyphenyl)thiazol-2-yl)phthalazin-1(2H)-one* (27c)

^1^H NMR (400 MHz, DMSO-*d*_6_): δ 8.84 (s, 1H), 8.43 (d, *J* = 8.0 Hz, 1H), 8.11–8.04 (m, 3H), 7.99 (t, *J* = 7.6 Hz, 1H), 7.61 (d, *J* = 7.6 Hz, 1H), 7.56 (s, 1H), 7.39 (t, *J* = 8.0 Hz, 1H), 6.95 (d, *J* = 8.4 Hz, 1H), 3.84 (s, 1H). ^13^C NMR (100 MHz, CDCl_3_): 159.9, 158.3, 157.4, 151.3, 140.6, 135.7, 134.4, 132.7, 129.7, 128.6, 127.4, 127.3, 126.8, 119.2, 114.1, 111.9, 111.9, 55.4. HRMS (ESI) calcd for C_18_H_14_N_3_O_2_S [M + H]^+^ 336.0807, found 336.0809.

#### *2-(2-(1-oxophthalazin-2(1H)-yl)thiazol-4-yl)benzamide* (28c)

^1^H NMR (400 MHz, DMSO-*d*_6_): δ 8.84 (s, 1H), 8.52 (s, 1H), 8.43 (d, *J* = 7.2 Hz, 1H), 8.16–7.98 (m, 6H), 7.86 (d, *J* = 6.0 Hz, 1H), 7.55 (s, 1H), 7.46 (s, 1H). ^13^C NMR (100 MHz, DMSO-*d*_6_): 168.3, 158.4, 157.7, 149.8, 141.5, 135.4, 135.3, 134.6, 133.5, 129.2, 129.0, 128.9, 128.1, 127.4, 127.0, 125.7, 113.8. HRMS (ESI) calcd for C_18_H_12_N_4_O_2_SNa [M + Na]^+^ 371.0579, found 371.0575.

#### *2-(4-(p-tolyl)thiazol-2-yl)phthalazin-1(2H)-one* (29c)

^1^HNMR (400 MHz, DMSO-*d*_6_): δ 8.82 (s, 1H), 8.42 (d, *J* = 8.0 Hz, 1H), 8.08 (t, *J* = 7.6 Hz, 1H), 8.05 (t, *J* = 7.6 Hz, 1H), 7.99 (d, *J* = 7.6 Hz, 1H), 7.95 (s, 1H), 7.90 (d, *J* = 8.0 Hz, 2H), 7.27 (d, *J* = 7.6 Hz, 2H), 2.35 (s, 3H). ^13^C NMR (100 MHz, DMSO-*d*_6_): 158.3, 157.3, 151.5, 140.6, 138.0, 134.3, 132.7, 131.6, 129.3, 128.6, 127.4, 127.3, 126.8, 126.4, 110.8. HRMS (ESI) calcd for C_18_H_14_N_3_OS [M + H]^+^ 320.0858, found 320.0862.

#### *2-(4-(2,5-dichlorophenyl)thiazol-2-yl)phthalazin-1(2H)-one* (30c)

^1^H NMR (400 MHz, DMSO-*d*_6_): δ 8.84 (s, 1H), 8.44 (d, *J* = 7.6 Hz, 1H), 8.17 (s, 1H), 8.12–7.98 (m, 4H), 7.64 (d, *J* = 7.6 Hz, 1H), 7.51 (d, *J* = 8.4 Hz, 1H). ^13^C NMR (100 MHz, CDCl_3_): 158.4, 156.7, 146.5, 140.9, 134.7, 134.5, 132.9, 132.9, 131.5, 131.3, 130.4, 129.0, 128.7, 127.5, 127.2, 126.9, 117.4. HRMS (ESI) calcd for C_17_H_9_N_3_ NaSCl_2_ [M + Na]^+^ 395.9741, found 395.9745.

#### *2-(4-(naphthalen-2-yl)thiazol-2-yl)phthalazin-1(2H)-one* (31c)

^1^H NMR (400 MHz, DMSO-*d*_6_): δ 8.89 (s, 1H), 8.59 (s, 1H), 8.46 (d, *J* = 8.0 Hz, 1H), 8.20–8.02 (m, 7H), 7.96 (d, *J* = 7.2 Hz, 1H), 7.59–7.53 (m, 2H). ^13^C NMR (100 MHz, CDCl_3_): 158.4, 157.6, 151.4, 140.8, 134.4, 133.6, 133.2, 132.8, 131.6, 128.7, 128.6, 128.3, 127.7, 127.5, 127.3, 126.9, 126.3, 126.1, 125.7, 124.4, 112.0. HRMS (ESI) calcd for C_21_H_13_N_3_OSNa [M + Na]^+^ 378.0677, found 378.0674.

#### *2-(4-([1,1’-biphenyl]-4-yl)thiazol-2-yl)phthalazin-1(2H)-one* (32c)

^1^H NMR (400 MHz, CDCl_3_): δ 8.57 (s, 1H), 8.56 (d, *J* = 6.8 Hz, 1H), 8.05 (d, *J* = 7.6 Hz, 2H), 7.91 (t, *J* = 7.2 Hz, 1H), 7.86 (t, *J* = 7.6 Hz, 1H), 7.82 (d, *J* = 7.6 Hz, 1H), 7.67 (d, *J* = 8.4 Hz, 2H), 7.65 (d, *J* = 7.2 Hz, 2H), 7.48 (s, 1H), 7.45 (t, *J* = 8.0 Hz, 2H), 7.35 (t, *J* = 7.6 Hz, 1H). ^13^C NMR (100 MHz, CDCl_3_): 158.4, 157.6, 151.1, 140.8, 140.8, 140.7, 134.4, 133.4, 132.8, 128.8, 128.7, 127.5, 127.4, 127.4, 127.1, 127.0, 126.9, 111.6. HRMS (ESI) calcd for C_23_H_16_N_3_OS [M + H]^+^ 382.1014, found 382.1017.

#### *5-amino-2-(4-(2-chlorophenyl)thiazol-2-yl)phthalazin-1(2H)-one* (25d)

^1^H NMR (400 MHz, DMSO-*d*_6_): δ 8.90 (s, 1H), 7.99 (s, 1H), 7.97 (dd, *J*_1_ = 7.6 Hz, *J*_2_ = 1.6 Hz, 1H), 7.59 (t, *J* = 8.0 Hz, 1H), 7.59 (dd, *J*_1_ = 8.0 Hz, *J*_2_ = 1.6 Hz, 1H), 7.53 (d, *J* = 7.2 Hz, 1H), 7.48 (t, *J* = 7.6 Hz, 1H), 7.42 (t, *J* = 8.0 Hz, 1H). 7.14 (dd, *J*_1_ = 8.0 Hz, *J*_2_ = 1.2 Hz, 1H), 6.66 (s, 2H). ^13^C NMR (100 MHz, DMSO-*d*_6_): 158.7, 156.9, 147.6, 146.8, 137.7, 134.2, 133.5, 132.0, 131.5, 130.8, 130.0, 127.9, 127.7, 119.3, 117.7, 113.6, 113.5. HRMS (ESI) calcd for C_17_H_11_N_4_OSClNa [M + Na]^+^ 377.0240, found 377.0240.

#### *(E)-2-((2-(4-(2-chlorophenyl)thiazol-2-yl)hydrazono)methyl)benzoic acid* (19)

mp 183.9–185.8 °C. ^1^H NMR (400 MHz, DMSO-*d*_6_): δ 12.80 (s, 1H), 8.84 (s, 1H), 8.01 (d, *J* = 7.6 Hz, 1H), 7.88 (t, *J* = 7.2 Hz, 2H), 7.63 (t, *J* = 7.2 Hz, 1H), 7.53 (dd, *J*_1_ = 7.6 Hz, *J*_2_ = 1.2 Hz, 1H), 7.48 (t, *J* = 7.2 Hz, 1H), 7.43 (t, *J* = 7.2 Hz, 1H), 7.41(t, *J* = 7.2 Hz, 1H ), 7.41 (s, 1H).^13^C NMR (100 MHz, DMSO-*d*_6_): 168.6, 167.8, 147.6, 140.8, 135.1, 133.7, 132.4, 131.6, 131.2, 130.8, 130.3, 129.5, 129.3, 127.7, 126.5, 109.3. HRMS (ESI) calcd for C_17_H_11_N_3_O_2_SCl [M − H]^−^ 356.0261, found 356.0261. Purity: 99.11% (*t*_R_ 13.58 min).

#### *(E)-2-((2-(4-(2-chlorophenyl)thiazol-2-yl)hydrazono)methyl)-6-fluorobenzoic acid* (24)

mp 211.2–211.7 °C. ^1^H NMR (400 MHz, DMSO-*d*_6_): δ 12.40 (s, 1H), 8.18 (s, 1H), 7.86 (dd, *J*_1_ = 7.6 Hz, *J*_2_ = 1.6 Hz, 1H), 7.69 (d, *J* = 8.0 Hz, 1H), 7.55–7.50 (m, 2H), 7.41 (t, *J* = 7.6 Hz, 1H), 7.37 (s, 1H), 7.35 (t, *J* = 7.6 Hz, 1H), 7.29(t, *J* = 8.8 Hz, 1H ). ^13^C NMR (100 MHz, DMSO-*d*_6_): 167.6, 166.1, 160.5, 147.6, 138.2, 138.1, 133.9, 133.8, 133.7, 131.5, 131.2, 130.8, 129.5, 127.7, 122.0, 116.5, 109.6. HRMS (ESI) calcd for C_17_H_10_N_3_O_2_SClF [M − H]^−^ 374.0166, found 374.0164. Purity: 97.09% (t_R_ 12.05 min).

#### *(E)-3-amino-2-((2-(4-(2-chlorophenyl)thiazol-2-yl)hydrazono)methyl)benzoic acid* (25)

mp 220.9–221.6 °C. ^1^H NMR (400 MHz, DMSO-*d*_6_): δ 12.8 (s, 1H), 11.1 (s, 1H), 7.93 (s, 1H), 7.89 (d, *J* = 7.2 Hz, 1H), 7.62 (d, *J* = 7.6 Hz, 1H), 7.52–7.43(m, 3H), 7.12 (d, *J* = 7.2 Hz, 1H), 7.08 (d, *J* = 8.4 Hz, 1H), 6.77 (s, 2H). ^13^C NMR (100 MHz, DMSO-*d*_6_): δ 165.8, 155.2, 154.3, 147.1, 146.3, 134.0, 132.7, 131.7, 131.5, 131.1, 130.3, 129.7, 128.1, 121.3, 114.5, 113.1, 111.6. Purity: 97.06% (*t*_R_ 19.58 min).

#### *(E)-2-((2-(4-phenylthiazol-2-yl)hydrazono)methyl)benzoic acid* (26)

mp 175.4–176.4 °C. ^1^H NMR (400 MHz, DMSO-*d*_6_): δ 12.80 (s, 1H), 8.84 (s, 1H), 7.99 (d, *J* = 7.6 Hz, 1H), 7.89–7.85 (m, 3H), 7.62 (t, *J* = 7.2 Hz, 1H), 7.47 (t, *J* = 7.6 Hz, 1H), 7.41 (t, *J* = 7.6 Hz, 2H), 7.33 (s, 1H), 7.30 (t, *J* = 7.6 Hz, 1H). ^13^C NMR (100 MHz, DMSO-*d*_6_): 168.6, 151.1, 140.8, 135.1, 135.0, 132.3, 130.8, 130.5, 129.3, 129.1, 128.0, 126.4, 126.0, 104.3. HRMS (ESI) calcd for C_17_H_12_N_3_O_2_S [M-H]^-^ 322.0650, found 322.0653. Purity: 95.72% (*t*_R_ 9.27 min).

#### *(E)-2-((2-(4-(3-methoxyphenyl)thiazol-2-yl)hydrazono)methyl)benzoic acid* (27)

mp 167.3–167.9 °C. ^1^H NMR (400 MHz, DMSO-*d*_6_): δ 12.80 (s, 1H), 8.82 (s, 1H), 8.00 (d, *J* = 8.0 Hz, 1H), 7.88 (d, *J* = 7.6 Hz, 1H), 7.62 (t, *J* = 7.6 Hz, 1H), 7.49–7.43 (m, 3H), 7.37 (s, 1H), 7.32 (t, *J* = 8.0 Hz, 1H), 6.88 (dd, *J*_*1*_ = 8.4 Hz, *J*_2_ = 2.0 Hz, 1H), 3.80 (s, 3H). ^13^C NMR (100 MHz, DMSO-*d*_6_): 168.7, 168.5, 160.0, 150.9, 140.8, 136.5, 135.0, 132.4, 130.8, 130.4, 130.1, 129.3, 126.4, 118.4, 113.8, 111.3, 104.7, 55.5. HRMS (ESI) calcd for C_18_H_14_N_3_O_3_S [M − H]^−^ 352.0756, found 352.0754. Purity: 95.56% (t_R_ 7.94 min).

#### *(E)-2-((2-(4-(3-carbamoylphenyl)thiazol-2-yl)hydrazono)methyl)benzoic acid* (28)

mp 297.9–298.4 °C. ^1^H NMR (400 MHz, DMSO-*d*_6_): δ 12.20 (s, 1H), 8.83 (s, 1H), 8.39 (s, 1H), 8.05 (s, 1H), 8.00 (dd, *J*_1_ = 7.2 Hz, *J*_2_ = 3.2 Hz, 2H), 7.88 (d, *J* = 7.6 Hz, 1H), 7.80 (d, *J* = 7.6 Hz, 1H), 7.63 (t, *J* = 7.6 Hz, 1H), 7.51–7.46 (m, 2H), 7.41 (s, 2H). ^13^C NMR (100 MHz, DMSO-*d*_6_): 168.8, 168.7, 168.4, 150.6, 140.8, 135.2, 135.1, 135.0, 132.4, 130.8, 130.4, 129.3, 129.0, 128.6, 126.9, 126.5, 125.4, 105.0. HRMS (ESI) calcd for C_18_H_15_N_4_O_3_S [M + H]^+^ 367.0865, found 367.0863. Purity: 97.89% (*t*_R_ 7.832 min).

#### *(E)-2-((2-(4-(p-tolyl)thiazol-2-yl)hydrazono)methyl)benzoic acid* (29)

mp 225.5–226.1 °C. ^1^H NMR (400 MHz, DMSO-*d*_6_): δ 12.80 (s, 1H), 8.83 (s, 1H), 8.00 (d, *J* = 7.2 Hz, 1H), 7.88 (d, *J* = 7.6 Hz, 1H), 7.75 (d, *J* = 8.0 Hz, 2H), 7.62 (t, *J* = 7.6 Hz, 1H), 7.47 (t, *J* = 7.6 Hz, 1H), 7.25 (s, 1H), 7.21 (t, *J* = 8.0 Hz, 2H), 2.32 (s, 3H). ^13^C NMR (100 MHz, DMSO-*d*_6_): 168.7, 168.6, 151.1, 140.7, 137.3, 135.0, 132.5, 132.3, 130.8, 130.6, 129.6, 129.2, 126.4, 126.0, 103.4, 21.3. HRMS (ESI) calcd for C_18_H_14_N_3_O_2_S [M − H]^−^ 336.0807, found 336.0808. Purity: 96.31% (*t*_R_ 13.17 min).

#### *(E)-2-((2-(4-(2,5-dichlorophenyl)thiazol-2-yl)hydrazono)methyl)benzoic acid* (30)

mp 205.0–206.0 °C. ^1^H NMR (400 MHz, DMSO-*d*_6_): δ 12.20 (s, 1H), 8.84 (s, 1H), 7.99 (d, *J* = 7.2 Hz, 1H), 7.95 (d, *J* = 6.4 Hz, 1H), 7.88 (d, *J* = 8.0 Hz, 1H), 7.62 (t, *J* = 7.2 Hz, 1H), 7.57 (d, *J* = 8.4 Hz, 1H), 7.52 (s, 1H), 7.47 (t, *J* = 7.6 Hz, 1H), 7.41 (dd, *J*_1_ = 8.8 Hz, *J*_2_ = 2.8 Hz, 1H). ^13^C NMR (100 MHz, DMSO-*d*_6_): 168.7, 167.9, 146.1, 141.2, 135.0, 134.9, 132.7, 132.3, 132.3, 130.8, 130.7, 130.6, 129.7, 129.4, 129.0, 126.5, 110.6. HRMS (ESI) calcd for C_17_H_10_N_3_O_2_SCl_2_ [M − H]^−^ 389.9871, found 389.9871. Purity: 97.66% (*t*_R_ 9.88 min).

#### *(E)-2-((2-(4-(naphthalen-2-yl)thiazol-2-yl)hydrazono)methyl)benzoic acid* (31)

mp 238.1–239.4 °C. ^1^H NMR (400 MHz, DMSO-*d*_6_): δ 12.80 (s, 1H), 8.87 (s, 1H), 8.40 (s, 1H), 8.04–8.01 (m, 2H), 7.96–7.89 (m, 4H), 7.64 (t, *J* = 7.6 Hz, 1H), 7.54–7.46 (m, 4H). ^13^C NMR (100 MHz, DMSO-*d*_6_): 168.8, 168.7, 151.0, 140.8, 135.1, 133.7, 132.9, 132.6, 132.4, 130.9, 130.4, 129.3, 128.6, 128.6, 128.1, 126.9, 126.5, 124.6, 124.4, 105.2. HRMS (ESI) calcd for C_21_H_16_N_3_O_2_S [M + H]^+^ 374.0963, found 374.0959. Purity: 96.39% (*t*_R_ 19.11 min).

#### *(E)-2-((2-(4-([1,1′-biphenyl]-4-yl)thiazol-2-yl)hydrazono)methyl)benzoic acid* (32)

mp 227.1–228.1 °C. ^1^H NMR (400 MHz, DMSO-*d*_6_): δ 12.35 (s, 1H), 8.83 (s, 1H), 8.01 (d, *J* = 6.0 Hz, 1H), 7.96 (d, *J* = 6.8 Hz, 2H), 7.88 (d, *J* = 6.0 Hz, 1H), 7.74–7.71 (m, 4H), 7.64 (t, *J* = 6.4 Hz, 1H), 7.48 (t, *J* = 6.0 Hz, 3H), 7.42 (s, 1H), 7.38 (t, *J* = 6.0 Hz, 1H). ^13^C NMR (100 MHz, DMSO-*d*_6_): 169.3, 169.3, 151.4, 141.4, 140.8, 140.2, 135.8, 134.9, 133.1, 131.5, 131.0, 130.1, 130.0, 128.6, 128.0, 127.6, 127.3, 127.1, 105.3. HRMS (ESI) calcd for C_23_H_18_N_3_O_2_S [M + H]^+^ 400.1120, found 400.1114. Purity: 97.60% (*t*_R_ 9.16 min).

#### *(E)-methyl 2-((2-(4-(2-chlorophenyl)thiazol-2-yl)hydrazono)methyl)benzoate* (22)

mp 145.8–147.7 °C. ^1^H NMR (400 MHz, CDCl_3_): δ 8.57 (s, 1H), 8.11 (d, *J* = 8.0 Hz, 1H), 7.95 (d, *J* = 7.6 Hz, 1H), 7.85 (dd, *J*_1_ = 7.6 Hz, *J*_2_ = 1.6 Hz, 1H), 7.59 (t, *J* = 8.4 Hz, 1H), 7.43 (t, *J* = 7.6 Hz, 1H), 7.42 (t, *J* = 8.0 Hz, 1H), 7.28 (s, 1H), 7.27 (t, *J* = 8.0 Hz, 1H ), 7.19 (t, *J* = 8.0 Hz, 1H), 3.93 (s, 3H). ^13^C NMR (100 MHz, CDCl_3_): 167.9, 167.2, 146.4, 141.8, 134.8, 132.7, 132.2, 132.0, 131.0, 130.6, 130.5, 129.0, 128.9, 128.6, 127.2, 127.0, 108.7, 52.4. HRMS (ESI) calcd for C_18_H_15_N_3_O_2_SCl [M − H]^−^ 372.0574, found 372.0575. Purity: 98.10% (*t*_R_ 15.32 min).

#### *(E)-ethyl 2-((2-(4-(2-chlorophenyl)thiazol-2-yl)hydrazono)methyl)benzoate* (23)

mp 172.0–172.6 °C. ^1^H NMR (400 MHz, CDCl_3_): δ 8.47 (s, 1H), 8.08 (d, *J* = 8.0 Hz, 1H), 7.94 (d, *J* = 8.0 Hz, 1H), 7.84 (dd, *J*_1_ = 7.6 Hz, *J*_2_ = 1.6 Hz, 1H), 7.57 (t, *J* = 8.0 Hz, 1H), 7.44–7.39 (m, 2H), 7.24 (t, *J* = 7.6 Hz, 1H ), 7.20 (s, 1H), 7.14 (t, *J* = 8.0 Hz, 1H), 4.37 (q, *J* = 7.2 Hz, 2H ), 1.40 (t, *J* = 7.2Hz, 3H). ^13^C NMR (100 MHz, CDCl_3_): 167.9, 166.7, 147.3, 141.1, 135.0, 133.3, 132.0, 132.0, 131.1, 130.5, 130.4, 128.9, 128.7, 128.6, 127.1, 126.9, 108.9, 61.3, 14.2. HRMS (ESI) calcd for C_19_H_17_N_3_O_2_SCl [M − H]^−^ 386.0730, found 386.0729. Purity: 99.78% (*t*_R_ 20.22 min).

## Additional Information

**How to cite this article**: Li, S. *et al*. Rational Design of Benzylidenehydrazinyl-Substituted Thiazole Derivatives as Potent Inhibitors of Human Dihydroorotate Dehydrogenase with *in Vivo* Anti-arthritic Activity. *Sci. Rep*. **5**, 14836; doi: 10.1038/srep14836 (2015).

## Supplementary Material

Supplementary Information

## Figures and Tables

**Figure 1:**
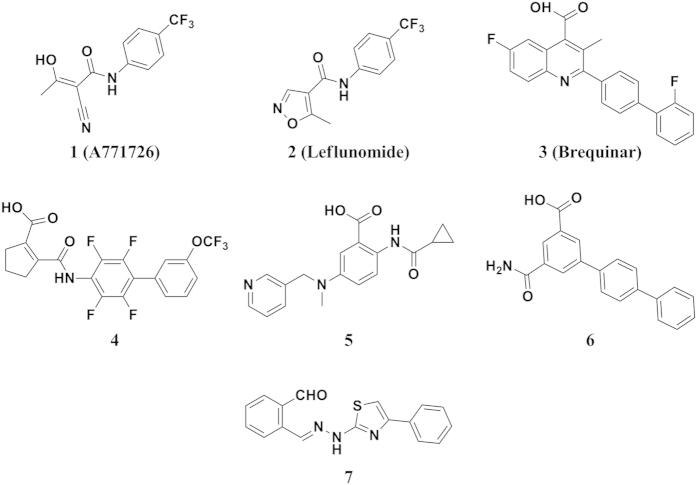
Selected structures of reported *h*DHODH inhibitors. Compound **7** is the lead in this study identified in our previous work.

**Table 1:**
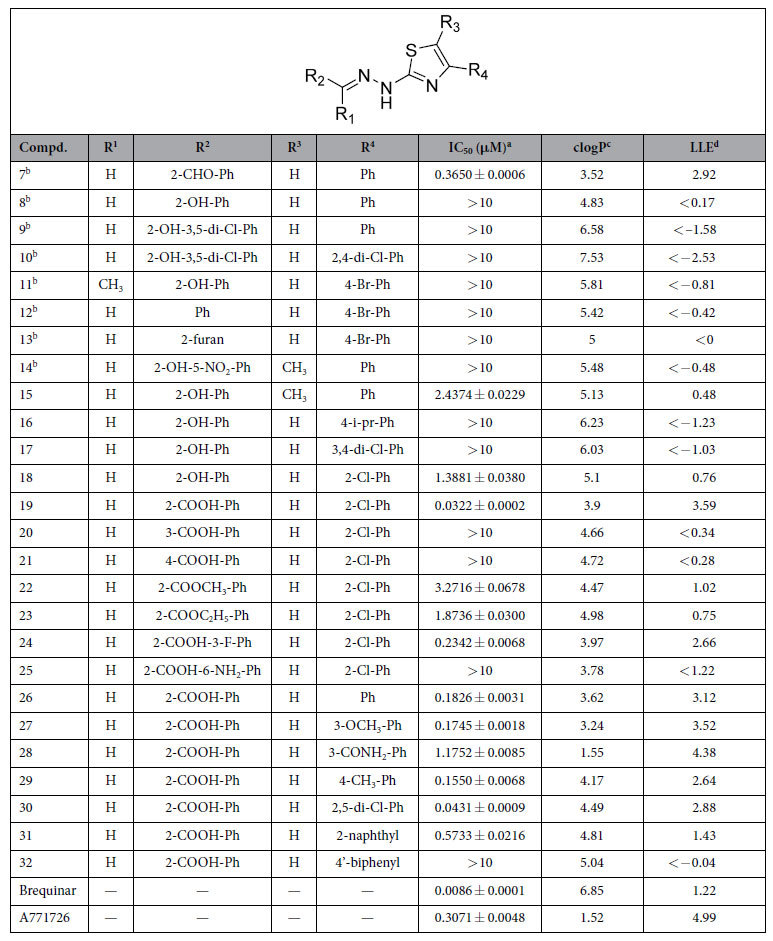
Chemical structure, ligand lipophilicity efficiency (LLE), and pharmacological activity of compounds 7–32.

^a^IC_50_ values were measured if the inhibition rate at 10 μM was larger than 50%. ^b^Compounds commercially obtained. ^c^clogP values were calculated by Qikprop software^62^. ^d^LLE values were calculated using the formula LLE = pIC_50_ − clogP^63^.

**Table 2:** Pharmacokinetic parameters of 19 after intravenous (IV) and oral (PO) administration.

Parameter^a^	19
IV dose (mg/kg)	1
*T*_1/2_ (h)	9.69 ± 3.15
AUC_0–∞_ (μg/L*h)	23318.81 ± 4501.24
Cl (L/h/kg)	0.04 ± 0.01
Vd_ss_ (L/kg)	0.35 ± 0.02
PO dose (mg/kg)	10
*T*_1/2_ (h)	8.20 ± 1.72
AUC_0–∞_ (μg/L*h)	53047.73 ± 11030.40
C_max_ (μg/L)	5310.50 ± 1910.47
*T*_max_ (h)	1.00 ± 0.87
*F* (%)	22.75 ± 4.73

^a^Compound was dosed to equal number of male Sprague-Dawley rats in IV and PO administration respectively (*n* = 3).

**Table 3:** Data collection and refinement statistics for the three *h*DHODH X-ray structures.

Inhibitor	7	19	27
Wavelengths (Å)	0.97852	0.97852	0.97852
Space group	P 3_2_ 2 1	P 3_2_ 2 1	P 3_2_ 2 1
Cell dimensions (Å)	90.530/90.530/123.820	90.860/90.860/122.940	90.390/90.390/122.900
Resolution (Å)	2.07	2.20	2.27
Number of reflections	36008	30357	27386
Redundancy	7.6(7.7)	8.8(9.0)	8.5(8.7)
Completeness (%)	99.6(99.9)	100.0(100.0)	99.9(100.0)
R_merge_ (%)	11.0(29.0)	14.9(36.7)	10.6(33.0)
I/σ (*I*)	14.8(8.3)	12.2(6.3)	18.9(9.4)
R/R_free_ (%)	15.1(17.2)	15.1(18.5)	15.1(19.0)
Bonds (Å)	0.007	0.007	0.007
Angles (deg.)	1.226	1.297	1.206
PDB ID code	4LS0	4LS1	4LS2
