# Supplementary material for: Rational Design of Benzylidenehydrazinyl-Substituted Thiazole Derivatives as Potent Inhibitors of Human Dihydroorotate Dehydrogenase with in Vivo Anti-arthritic Activity
Source: Sci Rep. 2015 Oct 7;5:14836. doi: 10.1038/srep14836 (PMC4595849; doi:10.1038/srep14836)
Supplement: Supplementary Information [file srep14836-s1.pdf]

Supporting Information

Rational Design of Benzylidenehydrazinyl-Substituted Thiazole Derivatives as Potent Inhibitors of Human Dihydroorotate Dehydrogenase with *in vivo* Anti-arthritic Activity

Shiliang Li[1, †], Guoqin Luan[1, †], Xiaoli Ren[1, †], Wenlin Song[1], Liuxin Xu[1], Minghao Xu[1], Junsheng Zhu[1], Dong Dong[1], Yanyan Diao[1], Xiaofeng Liu[1], Lili Zhu[1], Rui Wang[1], Zhenjiang Zhao[1, *], Yufang Xu[1, *], Honglin Li[1, *]

[1] *State Key Laboratory of Bioreactor Engineering, Shanghai Key Laboratory of New Drug Design, School of Pharmacy, East China University of Science and Technology, Shanghai 200237, China.*

[†] Authors contributed equally to this work

*To whom correspondence should be addressed. Email: zhjzhao@ecust.edu.cn, yfxu@ecust.edu.cn, hlli@ecust.edu.cn

Content

Table S1. HPLC analysis data of the commercially obtained compounds **7-14**............................S3
Table S2. Information about the 35 crystal structures of *h*DHODH. S5
Schemes S6

Scheme S1. Synthesis of the derivatives **15-18**, **20-21** S6

Scheme S2. Synthesis of the derivatives **19**, **24-32** S7

Scheme S3. Synthesis of the intermediates **24V-25V**...S8

Scheme S4. Synthesis of the derivatives **22-23** S9

Table S1. HPLC analysis data of the commercially obtained compounds **7-14**. The purity of the 8 bought compounds in the paper was determined by HPLC equipped with a Diamonsil-C18 column (250×4.6 mm, 5 μm particle size) and a UV/VIS detector setting of λ = 254 nm. All compounds were eluted with the two solvent systems (CH_3CN as organic phase in Method I and CH_3OH as organic phase in Method II) listed in the table at a flow rate of 1 mL/min.

Compound	Method I	Method II
**7**	CH_3CN:0.01M $(NH_4)_2CO_3$ = 50:50 (v/v)<br>retention time: 8.42 min<br>relative purity: 99.8%	CH_3OH:0.01M $(NH_4)_2CO_3$ =60:40 (v/v)<br>retention time: 5.72 min<br>relative purity: 99.9%
**8**	CH_3CN:0.01M $(NH_4)_2CO_3$ = 50:50 (v/v)<br>retention time: 18.11 min<br>relative purity: 97.7%	CH_3OH:0.01M $(NH_4)_2CO_3$ =98:2 (v/v)<br>retention time: 3.01 min<br>relative purity: 99.1%
**9**	CH_3CN:0.01M $(NH_4)_2CO_3$ = 50:50 (v/v)<br>retention time: 22.86 min<br>relative purity: 97.1%	CH_3OH:0.01M $(NH_4)_2CO_3$ =98:2 (v/v)<br>retention time: 3.72 min<br>relative purity: 97.3%
**10**	CH_3CN:0.01M $(NH_4)_2CO_3$ = 96:4 (v/v)<br>retention time: 5.15 min<br>relative purity: 99.9%	CH_3OH:0.01M $(NH_4)_2CO_3$ =98:2 (v/v)<br>retention time: 2.34min<br>relative purity: 99%
**11**	CH_3CN:0.01M $(NH_4)_2CO_3$ = 50:50 (v/v)<br>retention time: 21.47 min<br>relative purity: 94.0%	CH_3OH:0.01M $(NH_4)_2CO_3$ =98:2 (v/v)<br>retention time: 3.51 min<br>relative purity: 98.1%
**12**	CH_3CN:0.01M $(NH_4)_2CO_3$ = 50:50 (v/v)<br>retention time: 21.80 min<br>relative purity: 97.1%	CH_3OH:0.01M $(NH_4)_2CO_3$ =98:2 (v/v)<br>retention time: 3.55 min<br>relative purity: 96.5%
**13**	CH_3CN:0.01M $(NH_4)_2CO_3$ = 50:50 (v/v)<br>retention time: 18.24 min<br>relative purity: 95.4%	CH_3OH:0.01M $(NH_4)_2CO_3$ =98:2 (v/v)<br>retention time: 3.42 min<br>relative purity: 95.0%
**14**	CH_3CN:0.01M $(NH_4)_2CO_3$ = 50:50 (v/v)	CH_3OH:0.01M $(NH_4)_2CO_3$ =60:40 (v/v)

	retention time: 11.48 min<br>relative purity: 95.3%	retention time: 7.08 min<br>relative purity: 96.2%

Table S2. Information about the 35 crystal structures of *h*DHODH deposited in the Protein Data Bank.

No.	PDB ID	No. of buried water molecules in the ubiquinone-binding site	Resolution (Å)
1	1D3G	1	1.6
2	1D3H	2	1.8
3	2B0M	1	2.0
4	2BXV	1	2.15
5	2FPT	1	2.4
6	2FPV	1	1.8
7	2FPY	1	2.0
8	2FQI	1	1.95
9	2PRH	1	2.4
10	2PRL	1	2.1
11	2PRM	1	3.0
12	2WV8	1	1.9
13	3F1Q	2	2
14	3FJ6	3	1.8
15	3FJL	2	1.9
16	3G0U	2	2.0
17	3G0X	1	1.8
18	3KVJ	0	1.94
19	3KVK	2	2.05
20	3KVL	1	1.85
21	3KVM	2	2.0
22	3U2O	1	2.18
23	3ZWS	0	1.6
24	3ZWT	0	1.55
25	3W7R	0	1.68
26	4JTU	1	1.90
27	4OQV	2	1.23
28	4IGH	1	1.3
29	4JGD	2	2.05
30	4JS3	0	2.0
31	4JTS	0	2.21
32	4JTT	0	2.10
33	4LS0	3	2.07
34	4LS1	3	2.20
35	4LS2	2	2.27
total		43	

Schemes

Scheme S1. Synthesis of the benzylidenehydrazinyl-substituted thiazole derivatives **15-18**, **20-21** [1-3]

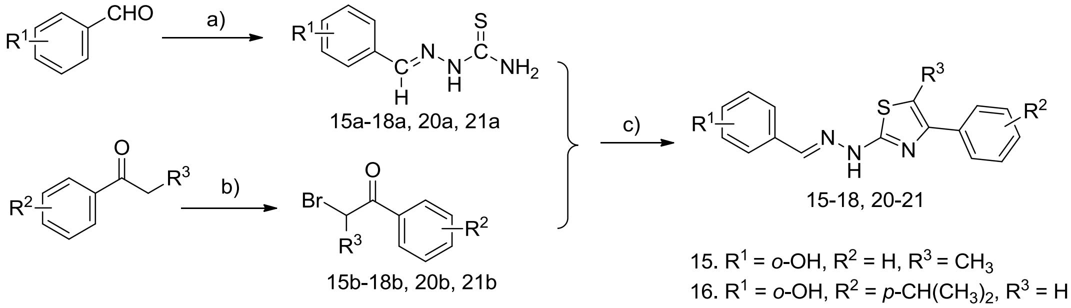

15. R^1 = *o*-OH, R^2 = H, R^3 = CH_3
16. R^1 = *o*-OH, R^2 = *p*-$CH(CH_3)_2$, R^3 = H
17. R^1 = *o*-OH, R^2 = 3,4-Cl, R^3 = H
18. R^1 = *o*-OH, R^2 = *o*-Cl, R^3 = H
20. R^1 = *m*-COOH, R^2 = *o*-Cl, R^3 = H
21. R^1 = *p*-COOH, R^2 = *o*-Cl, R^3 = H

Gerenal conditions: a) Thiosemicarbazide, ethanol/acetic acid, 40 ^oC 4-5 h; b) Br_2, $AlCl_3$, anhydrous ether, overnight at 0 ^oC; c) Ethanol, reflux for 3 h.

Scheme S2. Synthesis of the benzylidenehydrazinyl-substituted thiazole derivatives **19**, **24-32** [1,2,4,5]

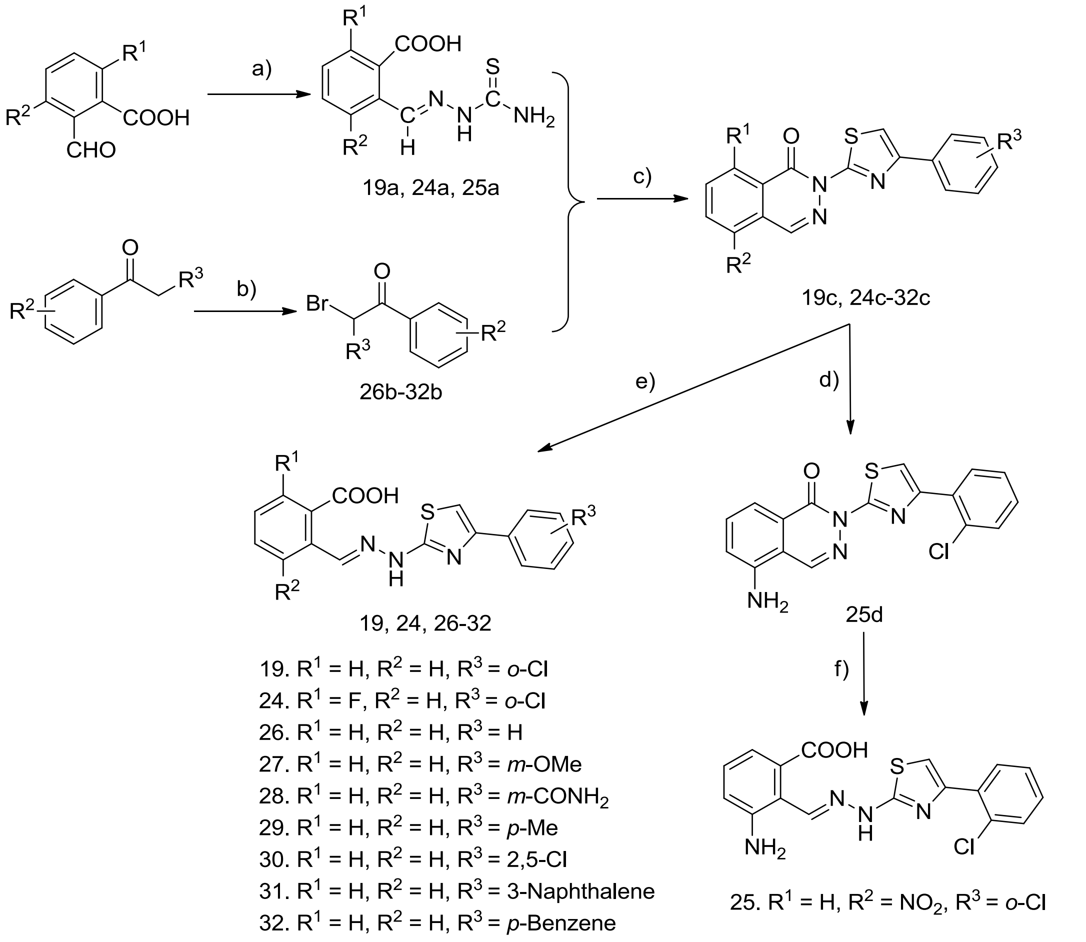

Gerenal conditions: a) Thiosemicarbazide, ethanol/acetic acid, 40 °C for 4-5 h; b) Br_2, $AlCl_3$, anhydrous ether, overnight at 0 °C; c) Ethanol, reflux for 3 h; d) $SnCl_2$, HCl, 80 °C for 5 h; e) NaOH, dry THF, overnight at rt; f) NaOH, dry THF, overnight at rt;

Scheme S3. Synthesis of the o-carboxybenzaldehyde intermediates **24V-25V** [6-12]

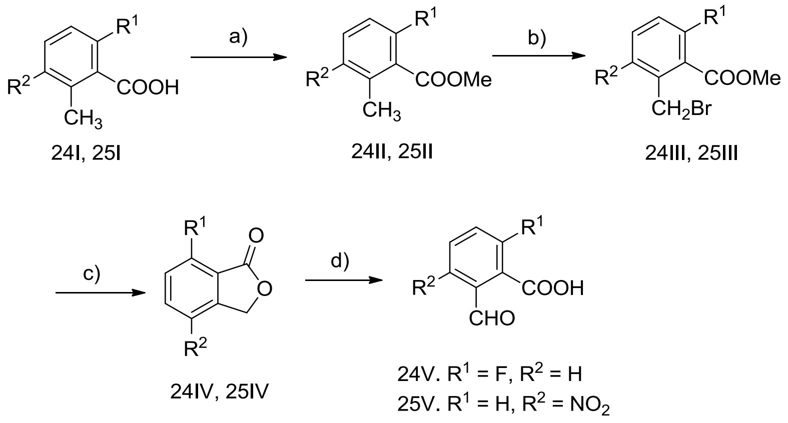

Gerenal conditions: a) 98% H_2SO_4, methanol, reflux overnight; b) NBS, AIBN, CCl_4, reflux 4-6 h; c) $CaCO_3$, H_2O/1,4-dioxane (1:1, v/v), 90 °C 5 h; d) 1. NBS, AIBN, $ClCH_2CH_2Cl$, reflux 4 h; 2. H_2O, reflux 5-6 h.

Scheme S4. Synthesis of the benzylidenehydrazinyl-substituted thiazole derivatives **22-23** [2,3,13-15]

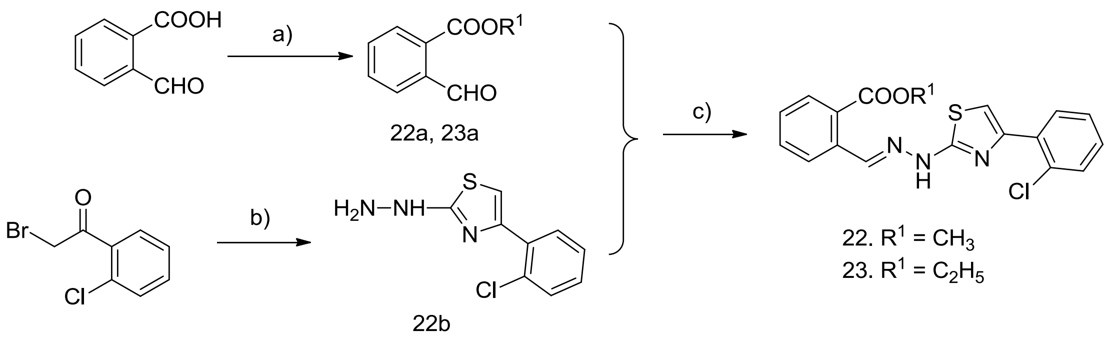

Gerenal conditions: a) Iodomethane/Iodoethane, potassium carbonate, dry DMF, reflux for 3 h; b) Thiosemicarbazide, 1,4-dioxane, overnight at rt; c) ethanol/acetic acid, rt for 5 h.

Reference

1. Bharti, S. K., Nath, G., Tilak, R. & Singh, S. Synthesis, anti-bacterial and anti-fungal activities of some novel Schiff bases containing 2, 4-disubstituted thiazole ring. *Eur. J. Med. Chem.* **45**, 651-660 (2010).

2. Sekimata, K. *et al.* A specific and potent inhibitor of brassinosteroid biosynthesis possessing a dioxolane ring. *J. Agric. Food Chem.* **50**, 3486-3490 (2002).

3. Porcal, W. *et al.* New trypanocidal hybrid compounds from the association of hydrazone moieties and benzofuroxan heterocycle. *Biorg. Med. Chem.* **16**, 6995-7004 (2008).

4. Furuta, T. *et al.* Synthesis of Axially Chiral Amino Acid and Amino Alcohols via Additive– Ligand-Free Pd-Catalyzed Domino Coupling Reaction and Subsequent Transformations of the Product Amidoaza [5] helicene. *J. Org. Chem.* **75**, 7010-7013 (2010).

5. Lipilin, D., Churakov, A., Strelenko, Y. A. & Tartakovsky, V. Selective reduction of the nitro group in the presence of the azoxy group. Synthesis of 2-(alkyl-NNO-azoxy) anilines. *Russ. Chem. Bull.* **51**, 311-318 (2002).

6. Mirabdolbaghi, R. & Dudding, T. An Indium-Mediated Allylative/Transesterification DFT-Directed Approach to Chiral C (3)-Functionalized Phthalides. *Org. Lett.* **14**, 3748-3751 (2012).

7. Koeberle, S. C. *et al.* Design, synthesis, and biological evaluation of novel disubstituted dibenzosuberones as highly potent and selective inhibitors of p38

mitogen activated protein kinase. *J. Med. Chem.* **55**, 5868-5877 (2012).

8. Zhang, S., Xu, L. & Trudell, M. L. Selective oxidation of benzylic alcohols and TBDMS ethers to carbonyl compounds with CrO3-H5IO6. *Synthesis* **2005**, 1757-1760 (2005).

9. Silva, N. M., Tributino, J. L., Miranda, A. L., Barreiro, E. J. & Fraga, C. A. New isoxazole derivatives designed as nicotinic acetylcholine receptor ligand candidates. *Eur. J. Med. Chem.* **37**, 163-170 (2002).

10. Xu, B. *et al.* < i> O</i>-Spiro< i> C</i>-aryl glucosides as novel sodium-dependent glucose co-transporter 2 (SGLT2) inhibitors. *Bioorg. Med. Chem. Lett.* **19**, 5632-5635 (2009).

11. van den Heuvel, M., van den Berg, T. A., Kellogg, R. M., Choma, C. T. & Feringa, B. L. Synthesis of a non-heme template for attaching four peptides: an approach to artificial iron (II)-containing peroxidases. *J. Org. Chem.* **69**, 250-262 (2004).

12. Liu, C., Lin, J., DeLucca, G. V., Douglas, B. G. & Liu, Q. (Google Patents, 2011).

13. Kiselev, E., Empey, N., Agama, K., Pommier, Y. & Cushman, M. Dibenzo [c, h][1, 5] naphthyridinediones as topoisomerase I inhibitors: design, synthesis, and biological evaluation. *J. Org. Chem.* **77**, 5167-5172 (2012).

14. Lata, D. *et al.* Thiosemicarbazone platinacycles with tertiary phosphines. Preparation of novel heterodinuclear platinum–tungsten complexes. *Polyhedron* **41**, 30-39 (2012).

15. London, I. M. *et al.* Regulation of protein synthesis. *The enzymes* **18**, 359-380 (1987).
